# Evaluation of the novel HEalthy Lifestyle Project (HELP) youth mental health e-intervention for lifestyle behaviour change and mental healthcare system impact: A randomized controlled trial protocol

**DOI:** 10.1371/journal.pone.0332363

**Published:** 2025-11-03

**Authors:** Ria Patel, Patricia E. Longmuir, Marjorie Robb, Natasha Baechler, Kimberly Courtney, Mark Norris, Gary Goldfield, Fiona Cooligan, Shannon Watson, Paula Cloutier, Clare Gray

**Affiliations:** 1 Children’s Hospital of Eastern Ontario Research Institute, Ottawa, Canada; 2 University of Ottawa, Faculty of Medicine, Ottawa, Canada; 3 Children’s Hospital of Eastern Ontario, Ottawa, Canada; University of Toronto, CANADA

## Abstract

**Background:**

Youth mental health is in crisis: suicide and self-harm doubled from 2019–2022; youth wait 8–12 months for care; 75% of youth with mental illness do not get care. Sleep, physical activity, and screen time impact mental wellbeing; lifestyle factors addressed with 95% of youth receiving mental health care. The virtual, asynchronous HELP (HEalthy Lifestyle Project) resource could enable step-by-step lifestyle behaviour change with or without professional support. HELP was collaboratively developed with youth experiencing mental distress, parents, and clinicians.

**Methods:**

Youth 12–17 seeking mental health services will be randomized to receive six months of immediate HELP e-resource access or a waitlist control (ClinicalTrials.gov, #NCT06232733). The primary outcome is emotional health (Strength and Difficulties Questionnaire total score). Secondary outcomes include behaviour change readiness, sleep, physical activity, screen time, and quality of life. All outcomes will be assessed at 0, 3, 6, and 12 months. Healthcare system utilization, extracted from health records at 18 months, will be compared relative to intervention access and compliance between participants and matched controls.

**Discussion:**

The HELP e-resource offers an accessible, stigma-free option for lifestyle support that could be widely available to youth experiencing mental distress. Healthy lifestyle behaviour change is a low-risk intervention that youth can access independently, or with support, whenever they are ready to engage, without waiting for specialist services. Healthy lifestyle behaviour change can enhance mental well-being and readiness to engage in treatment. Such changes could reduce the need for specialist support, alleviating an overburdened youth mental healthcare system.

## Introduction

Youth mental health is in crisis. Rates of youth suicide or self-harm doubled from 2019 to 2022 [1–3]. Youth with mental health concerns wait 8–12 months for care, with 75% of youth with mental illness unable to access care [1–3]. These delays increase suffering and prevent optimal outcomes. There is a clear need for novel, scalable mental health supports to meet this high demand.

Lifestyle behaviours, including sleep, physical activity, and screen time, are highly correlated with youth emotional and mental wellbeing [4–11]. These lifestyle behaviours are addressed with 95% of youth receiving mental healthcare (See S3 File Supplementary Material). Lifestyle behaviour change through intensive in-person support positively impacts mental health [5,6,9–11]. HELP (HEalthy Lifestyle Project) is a novel, virtual treatment strategy for supporting lifestyle behaviour change that targets the gap in timely youth mental healthcare. HELP engages youth in a step-by-step behaviour change journey focused on decreasing screen time, increasing physical activity, or improving sleep. This asynchronous, scalable support for self-guided lifestyle behaviour change starts at the first contact with the mental health system, so youth can benefit immediately—no waitlists.

HELP was designed collaboratively with over 70 youth, parents, and clinicians (Laurie et al, Manuscript in review). Pilot testing of the intervention suggested that for many youth, HELP was the first time they had been guided to take action on lifestyle behaviour change (Halle Smith et al, Manuscript in review); youth noted it as distinctly different from the educational interventions typically provided. In the pilot study, youth who engaged with HELP for three months demonstrated positive changes to emotional well-being and lifestyle behaviours.

Ensuring that youth are well rested, active, and have limited screen time could enhance their engagement in specialist services, potentially reducing the time that specialist support is required or even enabling youth to be supported by primary care providers. Reducing the time that specialists spend addressing lifestyle or the need for specialist referrals would enhance the mental healthcare system’s ability to support all youth with mental health challenges.

## Hypotheses

Providing HELP when youth (12–17 years) seek support will positively impact mental well-being by having the youth begin their wellness journey with positive lifestyle behaviour change. This will be evaluated based on the following:

1. PRIMARY: Youth engaging in the immediate intervention will have improved emotional health and enhanced lifestyle behaviours compared to those in the delayed intervention. Lifestyle changes will be sustained for an additional six months.
2. SECONDARY: Compared to a matched control group, those with HELP access will require less lifestyle support from mental healthcare professionals.

## Methods

### Guided by diverse lived experience

This research has been and continues to be guided by the lived experience of youth experiencing mental distress. The Lived Experience Advisory Group is a diverse team of ten youth and five parents who contributed to HELP development, study design, and knowledge translation plans. Members emphasized that the best way for youth to learn about the importance of lifestyle behaviours is through the mental healthcare system. Since that system is already stressed, they identified the need to evaluate the impact of HELP, not only on the participants, but on the youth mental healthcare system as well.

### Participants

#### Inclusion criteria.

Study participants will meet the following criteria:

a) Be 12–17 years of age;
b) Self-identify as experiencing emotional distress regardless of whether they are seeking, waiting for, or receiving support for emotional distress;
c) Able to provide informed consent to study participation;
d) Willing to be randomized to a study group;
e) Willing to complete objective behaviour measures if selected (one of three participants); and
f) Willing to provide consent for evaluation of mental healthcare system outcomes via their health record.

Study participants will be able to self-refer in response to public information about the study or can be referred by the care provider or community organization that they contact for support.

#### Exclusion criteria.

a) Unable or unwilling to complete questionnaires in English or French;
b) Unable or unwilling to use HELP in English (resources not yet translated);
c) Identified or suspected to have an eating disorder; these youth often have a compulsive exercise co-morbidity that may be negatively impacted by the physical activity promoting components within HELP; and
d) Health or family status deemed to be inappropriate for the study as per their most responsible clinician.

#### Recruitment.

1Call1Click is the centralized intake portal for all youth mental health services in the region. A best practice advisory flag has been implemented within the electronic health record system to identify potential study participants for intake staff. Intake staff will be educated on a standardized initial study screening process, including inclusion/exclusion criteria and consent. With permission, mental health clinicians can also refer youth for enrollment. Additional distribution of study material will be done via posters and social media posts in patient areas and through partner groups.

#### Consent.

The research coordinator will explain the study in full to the potential participant, who will then be given the opportunity to have all of their questions answered. If they agree to participate and meet the eligibility criteria, the researcher will obtain their consent to participate. Participants will choose the format to provide consent from three options: electronic (digital consent document, independent review of consent, archive and participant copy), verbal (phone or electronic connection, verbal review of consent, researcher records responses), or paper (review of consent with researcher, hard copy signature). Regardless of format, the researcher will review the study details, answer participants’ questions, confirm their identity, and ensure a copy of the completed consent form is provided to the participant.

#### Safety considerations.

There are no medical or health risks associated with participating in this study and participants will not be required to make any changes to their health services, supports, medications, or other treatments. Discomfort may occur when patients are answering questions about lifestyle behaviours that they think will be viewed negatively. The research coordinator will reassure participants that questions are asked only to compare to previous timepoints.

In cases where participation is triggering for a patient, the research coordinator will immediately notify the leading psychiatrist (CG) who will coordinate a clinical follow up as necessary. If a participant gives any indication that there is an increased risk of harm (suicidal ideation, self-harm, harm to others), the patient’s most responsible clinician will be contacted. Participants will be made aware of the limitations of confidentiality during the consent process.

#### Study design.

The study is a single-arm crossover randomized controlled trial. Youth will participate in the study for 12 months. Participants will be randomized (1:1 without stratification) to have access to the HELP e-intervention either immediately (between zero and six months) or after a delay of six months (between six and 12 months). This study design enables us to evaluate the impact of a delay in access to the intervention, to see how youth would progress with and without HELP access. The delayed intervention design also alleviates ethical concerns that would arise if some enrolled youth never had access to the study intervention. All participants will continue with standard care throughout the study. The 6-month intervention timeframe was chosen to expand on the pilot study which provided small but important benefits after only three months of intervention access. All study activities will be conducted remotely via digital surveys or mailed paper questionnaires. Completion of each questionnaire will require approximately one hour, working collaboratively with the research coordinator. In the pilot study, researcher support was needed to encourage engagement and questionnaire completion. The extent of this support will be tracked. All study measures will be administered by a researcher who is blind to group allocation. Outcomes will be assessed four times: at zero, three, six, and 12 months after enrollment.

#### Study outcomes.

The primary intervention efficacy outcome is youth emotional well-being as measured by the total scores of the full scale Strengths and Difficulties Questionnaire [12]. This instrument was selected because our target population is all youth experiencing mental distress, including those with relational or conduct problems. The HELP intervention does not target specific aspects of mental health, aligning with the broad screening aspect of the Strengths and Difficulties Questionnaire. A clinically significant improvement is defined as a score decrease of 15%, with the reduction occurring after participants have engaged with the intervention. This change is just under half of the Strengths and Difficulties Questionnaire improvement observed after six months of specialist community mental health services [13]. Emotional well-being is being used as an outcome as per the primary hypothesis correlating lifestyle behaviour change with improved mental health, as reflected in emotional wellbeing.

Secondary outcomes are measures of lifestyle choices (Youth Quality of Life-short form [14]), readiness for behaviour change (Stages of Change Questionnaire [15]), sleep hygiene (Adolescent Sleep Hygiene Scale [16]), daily physical activity (Habitual Activity Estimation Scale [17]), and leisure screen time (Adolescent Sedentary Activity Questionnaire [18]). Behaviour change theory emphasizes that individuals move through a series of stages, with the most effective intervention differing for each stage [19]. Achieving Stages 3–5 is required for behaviour change to occur (Stages 1 & 2 are moving toward the decision to change behaviour). HELP was developed to support youth in all stages of change [20].

A randomly selected sub-sample of participants (one in three enrolled) will wear a tri-axial accelerometer on a waist-worn belt for seven days after completing the questionnaires to objectively measure physical activity and sleep [21]. Sleep times, device removal times, and social media time will be recorded on a daily log sheet by these participants. In total, this additional data from ~40 participants will provide sufficient statistical power to evaluate the validity of the self-reported behavior data.

The healthcare system outcomes will be evaluated through youth mental healthcare system utilization. Whether HELP resource availability reduces mental health system visits or decreases the time that mental health specialists spend on lifestyle counselling will be assessed after study completion. These data will be collected from study enrollment for a period of 18 months. Quantitative measures include:

- Number of supports contacted;
- Types of support contacted (community provider, peer support, outpatient, day treatment, inpatient, etc.);
- Type of system contacts (request for support, appointment scheduling, treatment visit)
- Purpose of support (assessment, counselling, etc.);
- Type of mental distress;
- Professional seen (psychiatrist, psychologist, social worker, nurse, etc.); and
- Length of visit in minutes.

In addition, the complete text from each clinic visit note for each contact will be de-identified and retained in the study database for qualitative analyses to understand the lifestyle content and intent of documented discussions.

#### Sample size determination.

In the pilot study, youth engagement and full questionnaire completion was over 80% with assistance from a research coordinator. Since this trial will use the same supported method for questionnaire completion, the expected participation is that >80% of enrolled youth will have complete data. Complete data for 122 youth would provide 80% power to detect a 3.4-point difference in emotional health as measured by the Strengths and Difficulties Questionnaire total score. Based on the pilot data, a 3.4-point improvement in score would move the average score for study participants below the level of clinical concern and would be equivalent to the 19.5% improvement observed in the three-month pilot study. Given the pilot study data, the standardized effect size is hypothesized to be d = 0.5.

Gender and age-specific sampling will be extrapolated from the data depending on final recruitment demographics

#### Sex and gender.

Sex and gender are key factors in examining lifestyle needs as sleep, physical activity, and screen time are known to vary by these factors in adolescence [21–23]. Youth will be asked to self-report their gender identity and biological sex at birth; the impact of these factors will be examined in all analyses. Recruitment by sex and gender (boy, girl, non-binary, transgender) will be monitored and recruitment strategies will be adapted as needed. The Lived Experience Advisory Group includes gender diverse youth who have guided e-resource development and will continue to support the recruitment strategies they developed.

#### Participation timeline.

**Fig 1:**
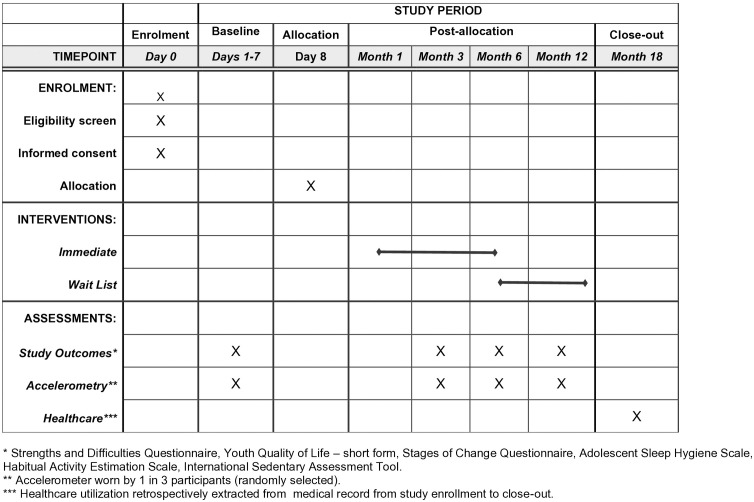
SPIRIT diagram of participant research participation and data analysis timeline. Fig 1 footnote: A-1,2,3,4: Assessments including questionnaires and additional accelerometry, sleep, screen time tracking for one in three youth. HA: HELP access including e-resource and kinesiologist support. HCR: Health Chart Review of patient records.

Study enrollment is planned from April 2025 to March 2026. Data collection continues for 1 year after study enrollment and is therefore expected to continue until March 2027, with results anticipated by the end of 2027. Refer to Fig 1 for details of the timeline for enrolled participants.

#### Intervention.

Research coordinators will directly engage with enrolled youth two to three times per week until they can continue the intervention independently. The initial session will introduce the participant to the HELP e-intervention (S1–S4 File Supplementary Material), review their baseline assessment results and focus their planned activities in light of their readiness for behaviour change. Subsequent sessions with the research coordinator will be scheduled as required by each youth in order for them to engage with the HELP e-resources on a consistent basis. Once consistent, independent engagement is established, formal sessions with the research coordinator will occur only when requested by the youth, reinforcing their autonomy to successfully change their behaviour. The HELP e-intervention resources are presented in four sections: Know Your Habits, Sleep, Screen Time, and Physical Activity. Know Your Habits provides youth with an interpretation of their personal baseline questionnaire results. The interpretation provides feedback on their current sleep, screen time and physical activity behaviours and makes recommendations for use of the HELP e-resources based on their current habits and readiness for behaviour change. Access to all HELP modules is always available, but Know Your Habits suggests personalized avenues for change based on each participant’s assessment results.

The Sleep, Screen Time and Physical Activity behaviour sections each have four sub-sections (Learn, Pros and Cons, Goals, and Resources), designed to align with the precontemplation, contemplation, preparation and action stages of behaviour change [19]. Learn conveys information linking the lifestyle behaviour and mental health. This section targets youth not yet considering behaviour change. Pros and Cons supports youth to consider the barriers and facilitators of behaviour change. This section guides youth to address any ambivalence as they contemplate making a change. The Goals section enables youth to choose from over 70 change plans that each provide a step-by-step process for making a small change to current behaviour. Each plan has a sequence of six to eight self-guided steps that youth complete in order to achieve their behaviour change goal. Each step typically requires two to 30 minutes to complete over one or more days. Roadblock activities are available to support the maintenance of recent behaviour changes. Information is also provided for setting SMART (specific, measurable, achievable, realistic, timely) goals. Youth can track their goals and accomplishments on the website. Resources provides additional links to external resources as well as access to the study kinesiologist.

Each participant will have a unique login from which intervention use (site visits, interactivity use, knowledge component completion) will be automatically tracked. This engagement data will also assess the frequency or type of intervention use, which will be considered when analyzing the intervention’s impact on any observed behaviour changes.

## Data analyses

### Data handling

Directly identifying information (name, phone number, email) will be recorded and used by the research coordinator to facilitate participant follow-up, support, and data completion. Indirectly identifying information (medical record number) will be used to retrospectively gather healthcare system outcomes. Sex and mental health diagnoses will be extracted from health records. All study information will be de-identified and coded with a unique participant ID. The principal investigator will retain a master list that links the participants’ code with their directly identifying information so data can be re-linked if necessary. Data cleaning will be on-going. Detailed analysis syntax and modeling will be retained for use in the final modeling when data collection is complete.

For direct measures of daily activity, monitoring data from at least three weekdays and one weekend day will be required. At least ten hours of daily accelerometer (Actigraph GT9X, Actigraph, Pensacola, FL) wear time during waking hours will be required to calculate daily physical activity [24]. ActiLife™ software (Actigraph, Pensacola, FL) calculates sleep duration based on measurement intervals lacking movement. Screentime will be measured by the social media tracking function in youths’ smartphones [25].

Upon study completion, de-identified study data will be available on request from the corresponding author with appropriate research ethics approvals.

### Analysis strategy

Changes between the immediate access and waitlist groups will evaluate HELP e-intervention efficacy. Differences between the six and 12-month assessments for the immediate access group will evaluate the sustainability of the intervention effects. Differences between the zero and six-month assessments for the waitlist group will evaluate changes in study outcomes over time without HELP. Comparisons between zero, three, and six-month outcomes will determine the required length of the e-intervention for optimal outcomes. Qualitative analyses will identify and evaluate changes in the need for support, potential use of lifestyle behaviours in treatment, and any events after HELP access that would be expected to change the required support.

### Quantitative analyses

Statistical significance will be p < 0.05 for all analyses. Correlations and paired t-tests will evaluate differences between the self-reported and objectively measured outcomes. The impact of sex will be investigated if sufficient youth do not identify as the gender aligned with their sex.

Repeated measures linear regression models will evaluate the changes to study outcomes from enrollment to the final assessment at 12 months. Patterns among those in the immediate versus delayed intervention will be compared to identify significant demographic variables (age, gender, sex, Indigeneity, type of mental health concern, type of support referral). Demographic variables that differ significantly between groups will be mandatory variables in the models of study outcomes. The primary analysis will be the change in Strengths and Difficulties score by changes in lifestyle habits, adjusted for age, gender, intervention adherence, and significant demographic variables. We will complete a mediation analysis of the impact of sleep, physical activity and screen time changes on the change in Strength and Difficulties score, creating a structural equation modeling path diagram.

For the healthcare system impact analysis, independent t-tests will compare the number of system contacts, service delivery visits and length of visits between groups. Chi-square statistics will compare type of system contacts, type of service delivery visit, professional seen, and purpose of service delivery visit between groups. Linear regression models will compare these outcomes, adjusting for significant demographic variables. Data loss is accounted for by the sample size as a significant outcome change (e.g., 50% reduction in mental health visits) would require a sample size of 98 (80% power, alpha = 0.05) which is equal to the sample size (122) at the minimum adherence as determined by the pilot study (>80%).

### Qualitative analysis

A content analysis of the qualitative data (i.e., healthcare visit documentation) will be completed to assess the impact of the HELP intervention on lifestyle counselling during mental health visits. The de-identified complete text of each mental healthcare visit clinic note will be reviewed. Each note will be coded to indicate the focus, content, information conveyed, and demographics (see S5: Supplementary Material). Descriptive statistics and a concept map will be used to summarize the data by age group (12–14, 15–17), sex, gender identity, and type of treatment. The goal of this analysis is to identify any trends suggesting variations in systematic lifestyle needs.

A thematic analysis of the clinic notes will also be completed using an inductive coding approach (no preconceived framework). This process will involve data immersion followed by analytical note and key phrase identification to enable initial code creation. Two members of the research team will both do a complete analysis and then the inductive codes will be reviewed and connected where appropriate. Final themes will be reviewed by the same demographics as listed above to identify trends.

## Discussion

### Potential problems

Through the 1Call1Click centralized intake, the research team will be able to approach all eligible and willing youth, minimizing recruitment biases. However, whether youth choose to enroll cannot be controlled. The age, gender, and initial intake reasons for youth who do and do not enroll will be evaluated to identify potential participant bias.

All efficacy outcomes rely on self-reported questionnaire data. Accelerometer measures of sleep and physical activity and smartphone records of social media use will be used to validate self-reported lifestyle behaviours. The blinded researcher conducting assessments will remind participants that there are no correct answers, but that the goal is to accurately record their current behaviours at the time of each assessment. The HELP intervention is only available via the website, so internet access is required.

Known limitations for the health system impact assessment include the willingness of participants to consent to the health record analysis and the variability and bias within health record notes and reporting practices of mental healthcare professionals. Differences in quantitative measures extracted from health records should not be influenced by individual practices; however, the extent to which lifestyle information is documented within these records may vary. The impact of this variation should be minimized by randomization.

### Knowledge translation

Youth-written project summaries, infographics, and videos will be distributed to all participating youth, partner organizations, and networks. Each youth will receive their own assessment outcomes and have access to overall study results. Participants will also be invited to contribute to the knowledge translation materials as this approach was found to be effective during the pilot study. Healthcare system results will be disseminated to professionals, families, policy makers, and hospital administrators. Academic presentations will target pediatricians and exercise medicine professionals. Two peer-reviewed papers regarding the results of this study will be published in open access journals for optimal research dissemination: one on the therapeutic impact of the intervention, and one on the impact of this new model of care for the healthcare system.

## Conclusion

The long-term goal of this study is to evaluate the physical and mental health benefits of healthy lifestyle behaviours to youth with mental health challenges by initiating an efficacy randomized controlled trial of the HELP intervention. However, intervention efficacy alone may not be sufficient to enhance lifestyle behaviour change treatment availability. The healthcare impact component of this study will define the impact of the HELP intervention on youth mental healthcare service delivery, enabling us to demonstrate both intervention efficacy and healthcare system benefits. The anticipated outcomes are evidence-based support for the HELP e-intervention in its ability to support sustainable lifestyle behavior change among youth seeking or receiving mental health support, in turn decreasing demand on the overburdened youth mental health care system.

## Supporting information

S1 File Completed SPIRIT checklist. (PDF)

S2 File Version of protocol approved by ethics committee at CHEO RI. (DOCX)

S3 File Supplementary Material. **HELP lifestyle interventions concept study.** LeBlanc JM, Norris M, S. Lee J, Gray C, Cloutier P, Robb M, al. Lifestyle issues are a frequent component of children’s mental health treatment: Retrospective data from a pediatric tertiary care center. Data is available from the corresponding author on request.
(DOCX)

S4 File HELP e-Intervention explanation. (PDF)
