# Supplementary material for: Evaluation of the novel HEalthy Lifestyle Project (HELP) youth mental health e-intervention for lifestyle behaviour change and mental healthcare system impact: A randomized controlled trial protocol
Source: PLoS One. 2025 Nov 3;20(11):e0332363. doi: 10.1371/journal.pone.0332363 (PMC12582452; doi:10.1371/journal.pone.0332363)
Supplement: S2 File — (DOCX) [file pone.0332363.s002.docx]

**PROTOCOL COVER PAGE**

**STUDY TITLE:** HELP (HEalthy Lifestyles Project) for Youth with Mental Distress E-Health Intervention: Patient and Healthcare Impacts

**PRINCIPAL INVESTIGATORS*:***

Dr. Clare Gray
Children’s Hospital of Eastern Ontario, CHEO Urgent Care
tel: 613-737-7600 x 2683; email: [gray_c@cheo.on.ca](mailto:gray_c@cheo.on.ca)

Dr. Pat Longmuir
Children’s Hospital of Eastern Ontario Research Institute, Ready, Set, Go
Tel: 613-737-7600 ext 3908; email: plongmuir@cheo.on.ca

**Co-Investigators*:***

Ms. Paula Cloutier
CHEO Research Institute, Mind Matters; pcloutier@cheo.on.ca

Dr. Mark Norris
CHEO, Eating Disorders and Medical Education; [mnorris@cheo.on.ca](mailto:mnorris@cheo.on.ca)

Dr. Marjorie Robb
CHEO, Mental Health; mrobb@cheo.on.ca

Dr. Gary Goldfield

CHEO Research Institute, Ready, Set, Go; ggoldfield@cheo.on.ca

Ms. Natasha Baechler
CHEO Research Institute, Family Advisor; natashabb2@icloud.com

Ms. Natasha McBrearty, Crossroads Mental Health; nmcbrearty@crossroadschildren.ca

Ms. Kimberly Courtney
CHEO Research Institute, Family Leader Program; kcourtney@cheo.on.ca

Ms. Fiona Cooligan
YouthNet / Réseau Ado; [fcooligan@cheo.on.ca](mailto:fcooligan@cheo.on.ca)
Ms. Shannon Watson
CHEO, Youth Forum; [swatson@cheo.on.ca](mailto:swatson@cheo.on.ca)

**Funded by***:* ***CHAMO grant funding has been confirmed for 2 years ($140,000). PSI Foundation grant is pending.***

Version date: 21-May-2024 Page **1** of **9**

# Background Information and Scientific Rationale

## Background and Rationale

### The Problem

500,000 Ontario youth (20-30%) have ≥ 1 mental health disorder^1^, 75% without specialist treatment^1^, compromising the timely care essential for optimal outcomes. 28,000 Ontario youth wait 8-30 months for care^2^. 200,000 youth have no access to care. Youth make 1.35 million out-patient and 70,000 emergency mental health visits/year. Youth suicidal ideation/self-harm doubled in 3 years^2^ prior to the pandemic. While mental health emergency hospitalizations skyrocket among youth; other causes are declining or stable. The critical shortage of mental health clinicians compounds increased treatment demand.

Youth are immersed in social media, doing little physical activity^3^ – lifestyle choices impacting their physical and mental health. Only 17.5% of Canadian youth meet sleep, physical activity and screen time guidelines^4^, and we know that meeting healthy lifestyle guidelines is associated with enhanced mental health. Achieving physical activity recommendations in grade 5 decreased adolescent mental illness risk by 47%^5^. Meeting screen time guidelines decreased mental health risk by 25%^5^. Each additional lifestyle guideline met further reduced mental health risk^6^.

### Summary of Work to Date

Our review of the medical records for 102 youth receiving specialist mental health support at CHEO found lifestyle counselling was included within mental health treatment for 95% of patients. In response, the expertise of 10 youth inpatients, 10 youth outpatients, 10 parents and 10 clinicians defined their desired type of lifestyle support. The HEalthy Lifestyle Project (HELP) resources were then iteratively and collaboratively designed with 12 inpatients, 33 outpatients, 18 parents, 11 clinicians and 23 Indigenous youth and elders (CHEO REB#20 62X).

Our pilot study of the HELP e-intervention enrolled youth contacting 1Call1Click or on the CHEO waitlist for specialist mental health services (CHEO REB#21 85X). 42% of youth waiting for specialist mental health support were eligible. Student researchers were able to approach 62% of eligible youth and successfully recruited 35% (n=10/29) in 4 months. Youth/families/clinicians requested the HELP pilot study be expanded to include youth seeking or receiving mental health support, adding new partners to our team (i.e., YouthNet/Réseau Ado, CHEO Youth Forum, 1Call1Click central intake). 26% (6/23) of teens enrolled in the next four months, with 5/6 youth having complete data. Most who expressed interest but did not enroll found the study questionnaires daunting as they were required to complete them virtually without researcher support. 83% (5/6) of enrolled youth demonstrated good engagement and measurable improvements in emotional health and lifestyle behaviours aligned with resource use. HELP resource engagement improved **
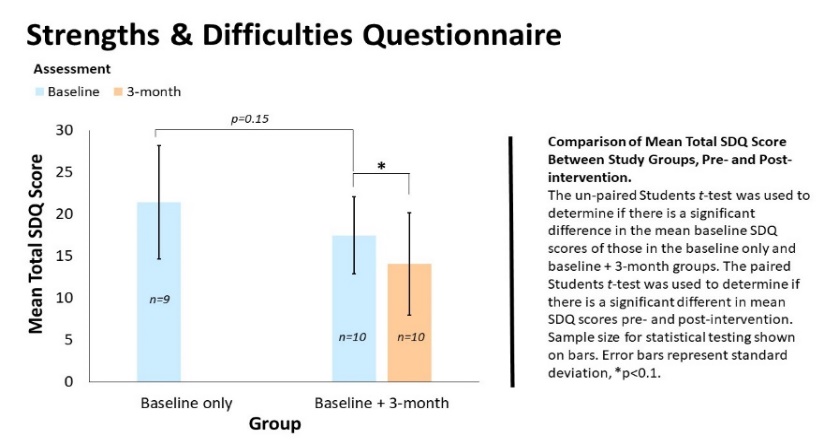
**sleep scores by 13%, decreased screen time by 0.96 hours/day and increased physical activity by 26 minutes/day. These changes allowed youth to achieve the recommended sleep and physical activity behaviours and reduce excessive screen use by approximately 50%.

Legend: Positive change was lower score for Strengths and Difficulties Questionnaire.

### Summary of Relevant Literature

**Children and Adolescents with Mental Illness have Unhealthy Lifestyles**

Child and adolescent psychiatrists routinely see patients whose unhealthy lifestyle choices significantly contribute to their presenting symptoms of mental distress. There is compelling evidence that poor sleep habits^10^, increased duration of screen time^11^, and poor nutrition^12^ are associated with worse mental health outcomes, including the development of depression and anxiety. Historic treatments failed to address these problems as their impact on mental health was significantly underestimated by health professionals, patients and families^13^.

**Lifestyle Changes Can Enhance Mental Health**

Research supports the effectiveness of lifestyle change for enhancing mental health. Lifestyle improvements associated with mental well-being include regular physical activity, balanced nutrition, getting enough sleep, decreased screen time, being outdoors, and making contributions in the service of others. Exercise can be as effective as psychotherapy and medication for treating some depressive disorders^14^; small changes in sleep duration can improve emotional regulation^15^; and nutritional interventions can improve overall functioning^12^. Increasing screen time is associated with decreased mental well-being and increased anxiety and depression in adolescents^16^. Furthermore, improvements to lifestyle behaviours can improve physical health, self-esteem, and quality of life^12,14,15,17–21^ and there is evidence that the positive effect may be additive^22.^.

**Rationale for Lifestyle Change interventions**

Youth are immersed in social media and doing little physical activity – poor lifestyle choices that impact their physical and mental health. Reviewing 102 health records of youth (50♀) receiving specialist support at CHEO we found few met criteria for major psychiatric diagnoses. Nevertheless, during the first two visits over 90% were counselled about changing sleep, physical activity and/or screen time. That mental health specialists are addressing the need for lifestyle change in the first visits with virtually all adolescents is evidence of the importance of lifestyle behaviour assessment/change as part of mental health treatment. There is a need to study lifestyle change intervention effectiveness in youth with mental illness because such interventions offer affordable, enjoyable, rewarding, and stigma-free treatment options that have no/few side effects/complications, and can be done individually or with family^14^. Healthy behaviours and moods spread through social networks, providing an opportunity for multiplier effects of lifestyle change on other family members, friends and neighbours^23^.

**Virtual Interventions to Support Lifestyle Change**

While the link between healthy lifestyle habits and mental health is well established, lifestyle change interventions have not been sufficiently studied in youth with significant mental health symptoms^13^. Through this project we will evaluate the HELP lifestyle change e-resource formats among youth experiencing mental distress. The HELP e-resources are available virtually to provide youth with access to these supports whenever they are ready to engage, even while they wait for specialist mental health treatment.

### Study Rationale

Lifestyle behaviour change through intensive in-person support has a positive impact on mental health. However, the youth mental health crisis creates a clear need to identify novel, scalable lifestyle behaviour change supports that can meet high demand. HELP is not an education intervention, but a novel virtual, asynchronous and scalable step-by-step support for self-guided lifestyle behaviour change. Youth anxiety/depression scores improved in 16 exercise RCTs^24^. 73 youth studies found improved psychological distress, anxiety, depression, and emotional disturbance linked to physical activity^25^. Decreasing recreational screen time, particularly phone/tablet use, improved adolescent mental health^26^. Sleep difficulties both determine and result from of youth mental health issues^27^. Targeting youth insomnia (6 weekly sessions) significantly improved psychological distress. These studies provide clear evidence of the mental health🡪lifestyle behaviour link^5,25,28–30^ and the impact of lifestyle behaviours on emotional health.

Knowing that only 17.5% of Canadian youth meet sleep, physical activity and screen time guidelines^31^, our team collaboratively developed the interactive HEalthy Lifestyle Project (HELP) e-intervention modules. HELP implements an innovative mental health care model that targets the important gap in timely youth mental health care. HELP is designed to engage youth in a step-by-step behaviour change journey focused on decreasing screen time, increasing physical activity, and/or improving sleep. We hypothesize that immediate access to the HELP intervention will support both prevention and treatment; reducing disease burden and long conventional therapy wait times^32^. The youth-initiated HELP format provides many youth-focused benefits^33^ and shares our lived experience of youth mental illness and mental health care, lifestyle behaviour change, and e-health expertise. Lifestyle e-interventions are stigma-free, have few side effects, can be done alone or with family and enable self-help strategies^14^. They also offer easy access, greater reach, flexibility for individual needs, and relatively lower cost.

## Study Objectives

This study will evaluate the efficacy of 6 months of HELP e-intervention access with kinesiologist support in a randomized controlled trial to determine if the changes in emotional health (primary outcome) and lifestyle behaviours (secondary outcomes) differ from changes among youth with delayed intervention access (control condition). Sustainability of the intervention will be assessed for an additional 6 months. Participants will be youth 12-17 years of age who contact youth mental health services (1Call1Click). We will also evaluate the impact of the HELP e-intervention on the use of mental healthcare services, the need for mental health professional support of lifestyle behaviours and the association between self-reported and objectively measured lifestyle behaviours.

### Research Questions

1. Do youth (12 to 17 years of age) who engage in the 6-month HELP e-intervention have a larger improvement in emotional health (measured by the Strengths and Difficulties Questionnaire) than youth who do not receive the intervention?
2. Does engagement in the HELP e-intervention improve lifestyle behaviour (physical activity, sleep or screen time)?
3. Are changes in emotional health and lifestyle behaviours sustained for at least 6 months after completion of the HELP e-intervention?
4. Do youth who engage in the 6-month HELP e-intervention utilize fewer mental healthcare resources, during and for 1 year following RCT participation, than youth who do not receive the intervention?
5. Do mental health professionals supporting youth who engage in the 6-month HELP e-intervention provide lifestyle behaviour support less frequently than for youth who do not receive the intervention?
6. Are self-reported (i.e., perceived) lifestyle behaviour changes (study questionnaire responses) aligned with objective measures of screen time (smartphone tracking) and sleep and physical activity (accelerometry) in a randomly selected sub-sample (1 of 3) enrolled participants?

We hypothesize that youth engaging in the HELP e-intervention will have improved emotional health and enhanced lifestyle behaviours in comparison to those receiving the delayed (post 6-months) intervention. We hypothesize that changes during the intervention will be maintained for an additional 6 months, and that self-reported measures will accurately reflect objective measurements. We hypothesize that youth engaging in the HELP e-intervention will require fewer youth mental health supports, and when supported will be less likely to require treatment directed at lifestyle behaviours.

# Eligibility Criteria

## Inclusion Criteria

Participants will be eligible for this study if they are 12-17 years of age when they are seeking, waiting for or receiving support for emotional distress. To be included in the study, youth must be: a) able to provide informed consent to study participation, b) able to engage in the HELP e-intervention in English (French translation of the e-modules will not be available until intervention efficacy is established), c) willing to be randomized to a study group, d) willing to complete objective behaviour measures if selected (1 of 3 participants), e) willing to complete the study questionnaires, and f) willing to provide consent for evaluation of mental healthcare system outcomes via their health record.

## Exclusion Criteria

Youth will be excluded if they have an identified or suspected eating disorder because these youth often have a compulsive exercise co-morbidity that may be negatively impacted by the physical activity promoting components of the HELP e-resources. Youth will also be excluded if their health or family status is deemed to be inappropriate for the study as per their most responsible clinician.

# Study Design

This research will investigate the patient and healthcare system impacts of the HEalthy Lifestyle Project (HELP) e-intervention. The research design is a randomized, controlled trial comparing youth who immediately receive the intervention to youth randomized to receive the intervention 6-months later (wait list control). Efficacy outcomes will be assessed at 0, 3, 6 and 12 months. Healthcare system impacts will be extracted from medical records from 0 to 18 months.

## Expected Duration of Participation

Youth will participate in the study for 12 months. All study visits and assessments are completed remotely. Youth can choose to complete the assessments via RedCAP survey or to have paper copies of the questionnaires sent by mail. The research coordinator will work virtually with each participant to support questionnaire completion. Completion of all questionnaires will require approximately 1 hour at each assessment timepoint. One of every three participants will be randomly selected to complete the objective behaviour measurements in addition to the questionnaires. Accelerometers will be distributed and collected by mail so that in-person visits to CHEO are not required. Participants selected for the objective measures will be required to wear an accelerometer on an elastic waistband 24 hours per day for 7 consecutive days. They will also be required to submit a screen shot of their social media screen time (app that already exists in smartphones) daily for the same 7 days. After the four assessment timepoints have been completed, youth will no longer be required to perform study activities but data collection on healthcare system impacts will continue to be collected via medical chart review for an additional 6 months. Participants randomized to the wait list control group will have access to the HELP e-intervention between the 6 and 12-month assessment timepoints.

| **Study Group** | **0 mos** | **0 to 3 mos** | **3 mos** | **3 to 6 mos** | **6 mos** | **6 to 12 mos** | **12 mos** | **12 to 18 mos** |
| --- | --- | --- | --- | --- | --- | --- | --- | --- |
| Intervention | Assess | HELP | Assess | HELP | Assess | ----- | Assess | Medical chart |
| Wait List Control | Assess | ----- | Assess | ----- | Assess | HELP | Assess | Medical chart |

Assess = completion of study questionnaires and objective measures (if applicable)
HELP = access to the HELP e-intervention with kinesiologist support
Medical chart = evaluation of mental health service use and inclusion of lifestyle behaviours in mental health service supports via medical chart review for 18 months from study enrollment

## Study Procedures/Evaluations

### Recruitment

The primary avenue for recruitment will be via 1Call1Click. During our pilot study of the HELP e-intervention (REB #21 85X) we developed a best practice advisory flag within EPIC (Appendix A) that was provided to 1Call1Click intake staff if the youth met the age criteria for the research study. Youth who access 1Call1Click have an initial assessment by intake staff. Staff have information about the research study and have been educated regarding the inclusion/exclusion criteria. If the research study could be appropriate for the patient, the intake staff will ask the patient/family if they would like more information about research. If the response is positive, intake staff will send an EPIC inbasket message to the research team who will follow up with the patient/family to discuss the study details.

During the pilot study we also received feedback from youth, families and clinicians requesting that the HELP e-intervention be more widely available to youth who are at various stages in their journey to mental wellness. Therefore, additional youth may be recruited through our two secondary recruitment options. CHEO mental health clinicians will be able to refer youth for enrollment in the research study if they have obtained permission from the youth to do so. This avenue for recruitment will enable youth on the wait list for specialist support or who are currently receiving CHEO support to be eligible for study recruitment. We will also distribute the study information via posters (Appendix B) and social media posts (Appendix C) in CHEO patient areas and through our youth partner groups (CHEO Youth Forum, CHEO-RI Family Leader Program, YouthNet / R$é$seau Ado, the study’s Lived Experience Advisory Group, etc.). Youth who contact the research team after viewing the study information will also be eligible to enroll.

### Consent

In keeping with the virtual design of the study, informed consent to participate will also be obtained electronically. Participants will be able to choose the format used to provide consent from the following options:

1. RedCAP e-consent
   RedCAP will be used to distribute the consent document, archive the complete e-consent and provide the youth with a copy of the completed consent. The researcher will verify the identity of the participant and their email address before sending the RedCAP link.
2. Verbal consent
   The researcher will connect with the participant by phone or electronically and verify their identity. The researcher will enter the participant responses during the consent discussion into RedCAP, which will automatically archive the completed consent and send a copy to the participant.
3. Paper consent
   Youth who prefer to complete a paper copy of the study consent will first speak with the researcher who will review the document, answer their questions and confirm their identity and how the consent form will be sent to the participant. The participant can choose to send/receive the paper consent form by email or regular mail.

If the patient is recruited through 1Call1Click or referral, the research coordinator will explain the study to the potential participant, including that the study materials (website, questionnaires) are only available in English at this time. The researcher will further explain that participation is voluntary and that the information will be recorded solely for research purposes and will be kept confidential. Finally, the research coordinator will explain that participation in the study will not change the mental health services available or the youth’s ability to access other services if desired. The participant will be given the opportunity to ask any questions that they wish regarding the study. Digital signatures will be acquired via RedCAP. Alternatively, participants may choose to provide a picture of a signed and dated written statement or an email confirming their consent to participate. Participants who are unable or unwilling to provide written consent have the option to provide verbal consent. All participants will receive a copy of the signed consent document.

If patients are recruited via our posters or social media posts, they will have the choice to either contact our research coordinator (and then follow the same process as for 1Call1Click described above) or to go directly to the virtual consent form on RedCap (via the published link or QR code). Informed consent will be obtained from youth able to consent for themselves (Appendix D). Youth who are unable to consent for themselves will be excluded as they are unlikely to be able to understand and/or engage independently in the HELP e-intervention. All REB-approved versions of the electronic informed consent will be archived and retained for auditing purposes. All youth who enroll in the study will be provided with the study resource document (Appendix N).

### Study Assessments

A researcher blinded to study group allocation will work with enrolled youth to complete all study assessments. The accurate/reliable study questionnaires include measures of emotional strength (Strengths and Difficulties Questionnaire)^34^, lifestyle choices (Youth Quality of Life-short form)^35^, readiness for behaviour change (Stages of Change Questionnaire^36^), and sleep (Adolescent Sleep Hygiene Scale)^37^, physical activity (Habitual Activity Estimation Scale)^38^, and leisure screen time (Adolescent Sedentary Activity Questionnaire)^39^ behaviours. All enrolled youth will complete all questionnaires at 0, 3, 6 and 12 months. As in our pilot study (REB #21 85X), the questionnaires (Appendix E) will be completed virtually using RedCAP, with automated scoring of the responses according to published methods. At each assessment, youth will be asked to voluntarily self-report age (years), gender (boy, girl, unkown, other, prefer not to answer), and their use of other mental health therapies (Appendix F). Study enrollment does not restrict access to or use of other mental health supports.

The blinded researcher will also administer the objective measures of sleep, physical activity and lifestyle to a randomly selected sub-sample of participants. One in every three participants will be provided by mail with an accelerometer to wear for 7 days at the time of the 0 and 6-month assessments. Each selected youth will wear a tri-axial accelerometer (Actigraph GT9X-BT, 180 day memory) on a waist-worn belt, over the right hip, in the mid axillary line, 24 hours/day for 7 days^40^ in addition to completing the study questionnaires. The goal is 24 hour accelerometer wear for 7 consecutive days to capture both physical activity and sleep, and to minimize data loss (monitor forgotten, etc.). The Actigraph GT9X-BT ([www.theactigraph.com](http://www.theactigraph.com)) can be worn for swimming or bathing to a 1-metre depth. Sleep times and reasons for device removal will be recorded on a log sheet (Appendix G). A minimum of 10 hours of wear time per day during waking hours will be required so that established cut points^41^ can be used to calculate daily minutes of sedentary, light and moderate+vigorous activity. The ActiLife software (Version 6.13.5, Actigraph Inc.) also calculates sleep duration based on measurement epochs lacking movement. At least 3 weekdays and 1 weekend day of valid data will be required. Screen use will be measured by utilizing the social media tracking function in the smartphone owned by each participant^42^. The researcher will explain to each participant how to access the social media tracking function that is build into their smartphone. Participants will be asked to take a screen shot of their social media use each day at the time they go to bed and to share the screen shot with the research team. Participants who do not own a smartphone will not complete the objective measures of lifestyle behaviours.

The research coordinator will collect the healthcare system impact outcomes via medical chart review after participants have completed the 12-month assessment. For the 18 months after study enrollment, we will record the number and type of youth mental healthcare system contacts (request for support, appointment scheduling, treatment visit). We will also track the number, type (e.g., community provider, peer support, outpatient, day treatment, inpatient), purpose (assessment, counselling), professional seen (e.g., psychiatrist, psychologist, social worker, nurse) and length (visit length in minutes) information for each service delivery visit (i.e., appointments/visits with any type of mental healthcare service provider). Health record notes for all mental healthcare visits will be reviewed in detail to identify all content related to sleep, physical activity or screen time assessments, discussions or recommendations for change. The complete text of each lifestyle-related clinic note will be de-identified for any patient-specific information before being transferred to our research database. Sex and mental health diagnoses will also be extracted from health records.

### HELP E-Intervention

All study participants will have access to the HELP e-intervention either immediately (HELP access between 0 and 6 months) or after a wait of 6 months (control condition, access between 6 and 12 months). The use of a wait list control is expected to decrease the impact of randomization on study recruitment and retention. Our unique HELP e-resources utilize age-appropriate graphics and interactive formats developed collaboratively with youth/parent/clinician partners. There are 4 sections: 1) Know Your Habits, 2) Physical Activity, 3) Screen Time, 4) Sleep. Know Your Habits provides youth with their personal baseline questionnaire results. Messages are tailored to each youth’s readiness for behaviour change and assessment results. Each behaviour section (physical activity, screen time, sleep) has 4 sub-sections. “Learn” conveys information linking the behaviour and youth mental health and targets youth not yet contemplating behaviour change. “Pros and Cons” targets ambivalence, supporting youth to consider the barriers/facilitators of behaviour change. “Goals” guides youth to choose from >70 step-by-step change plans. Each plan contains a sequence of 6-8 steps that youth complete to achieve their goal. Steps vary in required time (2-30 mins) and youth proceed through the steps at their preferred pace. SMART goal, overcoming road blocks and additional kinesiologist support resources are also provided. Youth can track their goals and accomplishments on the website. Access to all HELP e-modules is available to all youth but the website suggests individualized avenues for change based on each participant’s assessment results. Sample pages from the HELP e-intervention are provided in Appendix H.

Each participant will have a unique login to the HELP e-intervention (found at www.cheoactive.ca). By automatically tracking e-resource use (site visits, interactivity use, knowledge component completion), the website provides data regarding participant engagement with the intervention. These data also assess the frequency or type of e-resource use, important considerations in analyzing the intervention impact on behaviour change. Comparing initial and post-intervention questionnaire responses will indicate the potential emotional health/lifestyle behaviour change impacts of HELP e-resource access.

In our pilot study, youth who engaged with the HELP e-intervention demonstrated positive changes to emotional well-being and lifestyle behaviours. However, initiating routine engagement with the intervention was a struggle for a majority of participants. To facilitate their engagement into the study, the research coordinator will directly engage with enrolled youth 2-3 times/week until they are independently able to implement the intervention. Regular contacts with the research coordinator (via telephone, zoom or Facetime as per participant preference, Appendix I) throughout the 6-month intervention will support youth to engage with the intervention activities. The extent of this support provided to each participant will be tracked (# of sessions, length and content of each session).

### Additional Support

We do not expect study participation to cause significant psychological distress because the topics are limited to the health benefits of sleep, screen time and physical activity, rather than sensitive personal information. At the start of each assessment session, participants will be reminded that they can choose to answer or not answer each question, and that they can withdraw from the study at any time, even after the assessment or intervention has started. They will also be reminded that the information provided during the discussion is confidential for the research study, and will not be conveyed to the clinical care team except as required by law. Nevertheless, given the inherent vulnerability of this patient population we believe that it is essential to prepare in case study participation is triggering for a patient (e.g., a patient becomes upset or agitated). Should that occur, the research assistant or coordinator will follow the Safety Plan (Appendix J), during which Dr. Clare Gray will be notified immediately and she will coordinate the clinical follow up with the youth’s responsible clinician.

### Incidental Findings

Given the nature of the study, we do not anticipate that the study will produce incidental findings. However, in the event that a patient gives any indication that there is an increased risk of harm (suicidal ideation, self-harm, harm to others) during any study contact, Dr. Gray will be notified and she will communicate directly with the most responsible clinician for the participant. Dr. Gray will identify the most responsible clinician (physician or other healthcare professional) via the EPIC and 1Call1Click records. In the event that the participant does not have an identified care provider, Dr. Gray will follow up directly with the participant. Study participants will be made aware of the limitations of confidentiality regarding the information that they disclose to the research team through the consent process.

# Potential Risks & Benefits

## Potential Risks

There are no medical or health risks associated with participating in this study. Participants will not be required to make any changes to their health services, supports, medications or other treatments. The topics of the study (physical activity, sleep, and screen time) are not anticipated to cause harm. Discomforts during this study may occur when patients are answering questions about lifestyle behaviours that they consider or think will be viewed in a negative light. The research coordinator will reassure participants at the start of each assessment that the purpose of the questions is only to assess participants’ current behaviour so it can be compared to other study timepoints. The research coordiator will also emphasize that the research team recognizes that circumstances change regularly for all individuals. There will also be a risk to confidentiality if participants disclose information that, by law, must be reported.

## Potential Benefits to Society

The goal of this study is to enhance youth mental health through the innovative HELP e-intervention. Positive lifestyle behavior change among youth would be expected to decrease many health conditions associated with lack of sleep, excessive screen time or inactivity. We hypothesize that if youth begin important lifestyle changes that benefit their health, it will decrease their emotional distress and/or enhance their ability to participate in mental health interventions. This new model of patient care would therefore be expected to benefit healthcare delivery by providing more timely support to youth, encouraging positive health changes, enabling an evidence-based intervention to be provided for youth regardless of geographic location, fostering the support of youth mental health through primary care, and potentially decreasing the need for specialized mental health services.

## Potential Benefits to Participants

Participants in this study may benefit from having access to resources for healthy lifestyle behaviour change. Participating youth will be provided with documentation of the number of volunteer hours contributed to support their participation in this study (Appendix K). The number of hours will be determined based on each youth’s engagement with the intervention materials as reported during study contacts and tracked by the website. In addition, two hours will be allocated for the completion of each assessment (questionnaires), with an additional 10 hours if the participant completes the objective lifestyle measurements.

# Statistical Plan

## Sample Size Determination

The size of the study sample will be determined by the number of eligible youth contacts (with 1Call1Click or directly to the research team). Our pilot study approached 5-10 youth/week via 1Call1Click with 25% enrolling, providing an expected 400-800 potential and ~100-200 enrolled participants over 18 recruitment months. Complete data for 122 youth would provide 80% power to detect a 3.4 point difference in emotional health (Strengths & Difficulties Questionnaire), equivalent to the 19.5% improvement observed in our 3-month pilot study.

## Data Analyses

Data cleaning will be on-going throughout data collection. Initial data analyses of youth enrolled in the first 9 months of the RCT will be completed in months 15-20. Detailed analysis syntax and modeling will be retained for use in the final modeling/analyses when data collection is complete. Statistical significance will be set at p<0.05 for all analyses.

### Efficacy Outcomes

The primary outcome is youth emotional well-being as measured by the Strengths & Difficulties Questionnaire (SDQ^34^). We define a clinically significant improvement as a score decrease of 15%, with the reduction occurring only when participants have engaged with the intervention. This change is just less than 1/2 of the SDQ improvement observed after 6 months of specialist community mental health services^43^. We do not expect HELP benefits to be equivalent to traditional mental health interventions. However, we believe that the ability to improve emotional health half as well as traditional therapies would provide a meaningful and important change. In our pilot study, SDQ scores improved by 19.5% in 3 months among engaged youth. We will evaluate the change in SDQ from baseline to 6 months in a repeated measures model adjusted for age (years), gender, intervention adherence, type of mental health concern (e.g., anxiety, depression, etc. as documented during 1Call1Click intake), and support referral (community or specialist services recommended by 1Call1Click intake staff). The impact of sex will be investigated if participants include a sufficient number of youth who do not identify as the gender aligned with their sex.

Secondary efficacy outcomes are measures of readiness for behaviour change (i.e., stage of change) and sleep, physical activity and screen time behaviour. Behaviour change theory emphasizes that individuals move through a series of stages, with the most effective intervention differing for each stage. HELP supports youth in all stages of change. “Learn” targets youth not yet considering behaviour change. “Pro’s and Con’s” guides youth to address their ambivalence towards change. “Goals” provide step-by-step plans so youth can act on their intention to change behaviour. “Roadblocks” support the maintenance of recent behaviour changes. Paired t-tests will examine participants’ stage of change at enrollment and 6 months to assess whether HELP enhanced their readiness for behaviour change. Modeling how the behaviour change stage differs, with adjustments for age, gender, intervention adherence, type of mental health concern and support referral, will assess whether HELP similarly impacts behaviour change among all youth. The same analyses will be completed for each behaviour outcome with stage of change at 6-months a mandatory variable as theory indicates that achieving Stages 3 to 5 is required for behaviour change to occur (Stages 1 & 2 are moving toward the decision to change behaviour).

A repeated measures model of the primary and secondary outcomes will evaluate changes between study groups, comparing changes occurring during intervention access to changes when the intervention is not available. Changes between the 0 and 6-month assessments for youth randomized to immediate intervention access and between the 6 and 12-month assessments for youth randomized to the wait list group will evaluate HELP e-intervention efficacy. Differences between the 6 and 12-month assessments for youth randomized to immediate intervention access will evaluate the sustainability of the intervention effects. Differences between the 0 and 6-month assessments for youth randomized to the wait list group will evaluate changes in study outcomes over time without the HELP e-intervention. Comparisons between 0 and 3-month and 3 and 6-month outcomes will determine the required length of the HELP e-intervention for optimal outcomes. All models will be adjusted for age, gender, intervention adherence, type of mental health concern and support referral.

### Healthcare System Outcomes

Measures of youth mental healthcare system utilization will be compared between the intervention and wait list control participants. Independent t-tests will compare the number of system contacts, number of service delivery visits and length of service delivery visits for each timeframe (with/without intervention access). Chi-square statistics will compare categorical variables (type of system contacts, type of service delivery visit, professional seen, purpose of service delivery visit) between the intervention and control participants for each timeframe. Linear regression models will compare these outcomes between the intervention and control participants, adjusting for demographic variables (age, sex, mental health concern). Repeated measures models will evaluate the pattern of changes to mental health service use throughout study participation.

The frequency of lifestyle support during mental health services will be evaluated using mixed methods. Each comment in the research database will be coded to indicate the focus (assessment, discussion, recommendation), content and information conveyed by the comment utilizing the same procedures and coding framework developed during our retrospective chart review^44^. Descriptive statistics (frequency tabulations, means or medians (variance) as appropriate) are then used to summarize the data. We will also analyze these data qualitatively, using an inductive approach (no preconceived coding framework). Independent analyses of the qualitative data will be completed by two members of the research team to summarize the lifestyle-related discussions during patient visits. Inductive analyses will begin with data immersion, reading/re-reading the extracted comments. Analytical notes and key phrase identification will identify concepts and enable initial code creation (NVivo nodes). Analyses, interpretation and revisions will continue throughout the analytical process. Data will be interpreted relative to understanding lifestyle needs and counselling for these youth. Key findings will be reviewed separately by age group (12-14, 15-17), sex and type of treatment to identify any trends suggesting that lifestyle needs vary systematically.

## Protocol Deviations

Any and all deviations will be reported to the REB in a timely manner.

# Data Handling and Record Keeping

## Data Collection and Management

### Data Sources

The majority of the study data will come directly from the research participants via their responses to the study questionnaires and their self-reported age and gender. Data on participant engagement with the HELP e-intervention will be generated by the HELP website as the tracking of page views and goal setting activities. Healthcare system impact data will be gathered from participant medical records.

### Types of data (direct or indirect identifiers)

This study will utilize identifiable information. *Directly identifying information* (name, phone number, email) will be recorded to facilitate participant follow up and support. The research coordinator will utilize this information to deliver the intervention and schedule the study assessment visits. The indirectly identifying informaiton will be the EPIC MRN, which will be used to retrospectively gather the healthsystem impact outcomes.

*All other study information* (e.g., data captured on data collection tools, website tracking) *will be de-identified.* Direct and indirect identifiers will be removed and replaced with a unique ID code for each participant. The principal investigator will retain a master list that links the participants’ code with their directly identifying information so data can be re-linked if necessary.

## Data collection and review of data

### Administrative data: Master list

For the purposes of study administration, the CHEO research team will maintain a master list with direct identifiers. These include name, phone number and email. Each participant will be assigned a unique study ID that will be used on all data collection tools (e.g., case report forms, questionnaires).

### Study data

The data collection tools are the standardized questionnaires administered to assess intervention efficacy. Specifically, they are the measures of emotional strength (Strengths and Difficulties Questionnaire)^34^, lifestyle choices (Youth Quality of Life-short form)^35^, readiness for behaviour change (Stages of Change Questionnaire^36^), and sleep (Adolescent Sleep Hygiene Scale)^37^, physical activity (Habitual Activity Estimation Scale)^38^, and leisure screen time (Adolescent Sedentary Activity Questionnaire)^39^ behaviour. At each study assessment timepoint, participant’s self-reported age and gender will be recorded in RedCap, sample format is attached (Appendix F). The kinesiology support contact form (Appendix I) will standardize notes summarizing each support session or assessment delivered by a researcher. The retrospective collection of data from the medical records to analyze the healthcare system impacts will be completed using a RedCAP database (Appendix L).

## Data storage and retention

Administrative data will be stored only on the CHEO secure server in an Excel file that is password protected and accessible only to the study team.

Study data will be password-protected and stored on CHEO REDCap. REDCap (Research Electronic Data Capture) is a secure, web-based application designed exclusively to support data capture for research studies. The application and data are stored on CHEO servers. Local support for REDcap is provided by CHEO’s Clinical Research Unit Data exported onto an external drive for the purposes of analysis will be de-identified and stored with two locks of protection.

Paper study documentation is not expected unless a participant requests to complete study materials in that format. All paper study documentation will be stored in a locked cabinet in a locked office in Research Institute #1-212.

To maintain data security and participant confidentiality, administrative data and study data will always be stored separately. The study data and results will be maintained at CHEO until 7 years after all analyses have been completed and the results of the study have been published in appropriate peer-reviewed journals. After that time, all paper and electronic records from the study located at CHEO, including all back-up copies of the data, will be erased using approved protocols.

It is recognized that ensuring access to research data via secure data repositories is increasingly common. The study data will be stored in such a repository if required by CHEO Research Institute policy, or the policies of study funding organizations and peer-reviewed journals. The data thus deposited would continue to be available for use in other research as long it was maintained in the data repository.

## Data access

Access to all study documentation will be limited to members of the CHEO research team and approved authorities (e.g., Study Sponsor, Institution where study is conducted, REB of Record) for the purposes of study-related monitoring, audits and inspections. Source documents may contain identifiable data and will not be transferred externally.

## Data transfer

No data transfer is planned during this study.

## Futures uses of data/ Data Sharing

Data collected for this research may be used in future related research projects that are either an extension of the original project or in the same general area of research (secondary use of data). Researchers outside of this specific study may request access to the coded data for new research purposes. Participants will not be asked to provide additional informed consent for the use of the coded data for future research.

# Budget

Funding applications have been submitted to CHAMO and the PSI Foundation. The CHAMO application has been recommended for funding to the provincial oversight committee (final decision end of March 2024). The CHAMO funding will support the 6-month efficacy randomized controlled trial of the HELP e-intervention. The PSI Foundation application will fund the healthcare system impact, sustainability of the intervention and objective measures components of the study. Detailed budgets from the funding proposals are provided in Appendix M.

# Dissemination \ Publication Plan

Our equity diverse, Lived Experience Advisory Group (LEAG) is comprised of 10 youth and 5 parents, all of whom have lived experience of youth mental distress. The LEAG developed our KT plan and will continue to guide our KT materials/activities. The KT plan they developed includes Annual Grand Rounds presentations (Y1: study protocol, Y2: efficacy RCT data, Y3: healthcare system data) because youth tell us that informed clinicians are the most effective avenue for sharing study information. Youth-written project summaries, infographics and videos will be developed and distributed to all youth partner organizations and networks/sites. Our LEAG members will also distribute the study results via social media networks. Each youth will receive their own assessment outcomes and have access to study presentations/results via the Healthier CHEO Kids web site ([www.cheori.org](http://www.cheori.org)) and partner networks. We will also invite study participants to contribute to the KT materials/activities as we found this approach very effective during our pilot study of the HELP e-resources.

Healthcare system results will be disseminated to professionals, families, hospital administrators and policy makers via a Children’s Healthcare Canada webinar presentation. Academic presentations will target paediatricians (Canadian Paediatric Society conference presentation) and exercise medicine professionals (North American Society for Pediatric Exercise Medicine conference presentation). We will publish three papers from this study in peer-reviewed journals for optimal dissemination of our research to the academic community. In year 1, we will publish the study protocol. In year 3, we will publish two papers reporting the study results. One paper will focus on the therapeutic impact of the intervention. The second paper will focus on the impact of this new model of care for the healthcare system. All papers will be published in open access journals.

# References

1. Ministry of Children and Youth Services. A Shared Responsibility: Ontario’s Policy Framework for Child and Adolescent Mental Health [Internet]. Toronto, Ontario: 2006. Available from: http://www.children.gov.on.ca/htdocs/English/documents/specialneeds/mentalhealth/framework.pdf

2. Children’s Mental Health Ontario. Kids Can’t Wait [Internet]. 2020;Available from: https://cmho.org/wp-content/uploads/CMHO-Report-WaitTimes-2020.pdf

3. Paglia-Boak A, Hamilton HA, Adlaf EM, Beitchman J, Wolfe D, Mann RE. The mental health and well-being of Ontario students 1991-2013: Detailed OSDUHS findings. Toronto, Canada: 2015.

4. Roberts KC, Xiaoquan Y, Carson V, J-P C, Janssen I, M.S. T, Yao X, Carson V, Chaput JP, Janssen I, Tremblay MS, Xiaoquan Y, Carson V, J-P C, Janssen I, M.S. T, Yao X, Carson V, Chaput JP, Janssen I, Tremblay MS, Xiaoquan Y, Carson V, J-P C, Janssen I, M.S. T. Meeting the Canadian 24-hour movement guidelines for children and youth. *Heal Reports* [Internet]. 2017;28:3–7.

5. Loewen OK, Maximova K, Ekwaru JP, Faught EL, Asbridge M, Ohinmaa A, Veugelers PJ. Lifestyle Behavior and Mental Health in Early Adolescence. *Pediatrics* [Internet]. 2019;143:e20183307. Available from: https://publications.aap.org/pediatrics/article/77071

6. Sampasa-Kanyinga H, Colman I, Goldfield GS, Janssen I, Wang J, Podinic I, Tremblay MS, Saunders TJ, Sampson M, Chaput J-P. Combinations of physical activity, sedentary time, and sleep duration and their associations with depressive symptoms and other mental health problems in children and adolescents: a systematic review. *Int J Behav Nutr Phys Act* [Internet]. 2020;17:72. Available from: https://ijbnpa.biomedcentral.com/articles/10.1186/s12966-020-00976-x

7. Centre for Addiction and Mental Health. The Mental Health Crisis is Real [Internet]. 2009;Available from: www.camh.ca › driving-change › the-crisis-is-real

8. Ontario’s Mental Health and Addictions Leadership Advsiory Group. Mental Health and Addictions Realizing the Vision: Better Mental Health Means Better Health [Internet]. Toronto, Canada: 2017. Available from: https://www.health.gov.on.ca/en/common/ministry/publications/reports/bmhmbh_2017/vision_2017.pdf

9. Children’s Mental Health Ontario. Children and Youth Mental Health Survey: Getting Help in Ontario. 2017.

10. Owens J, Au R, Carskadon M, Millman R, Wolfson A, Braverman PK, Adelman WP, Breuner CC, Levine DA, Marcell A V., Murray PJ, O’Brien RF. Insufficient sleep in adolescents and young adults: An update on causes and consequences. *Pediatrics*. 2014;134:e921–e932.

11. Maras D, Flament MF, Murray M, Buchholz A, Henderson KA, Obeid N, Goldfield GS. Screen time is associated with depression and anxiety in Canadian youth. *Prev Med (Baltim)* [Internet]. 2015;73:133–138.

12. Calms J, Davison K, Grant-Moore J, Jaques M, Mallhot-Hall L, Ng E, Palmer J, Seely C, Sengmueller E. The Role of Nutrition Care for Mental Health Conditions [Internet]. 2012 [cited 2021 May 13];Available from: https://www.dietitians.ca/DietitiansOfCanada/media/Documents/Resources/Nutrition-and-Mental-Health-2.pdf?ext=.pdf

13. Walsh R. Lifestyle and Mental Health. *Am Psychol*. 2011;66:579–592.

14. Cooney GM, Dwan K, Greig CA, Lawlor DA, Rimer J, Waugh FR, McMurdo M, Mead GE. Exercise for depression. *Cochrane Database Syst Rev* [Internet]. 2013 [cited 2021 May 13];Available from: http://doi.wiley.com/10.1002/14651858.CD004366.pub6

15. Impact of Sleep Extension and Restriction on Children’ s Emotional Lability and Impulsivity. 2012;Available from: www.pediatrics.org/cgi/doi/10.1542/peds.2012-0564

16. Twenge JM, Campbell WK. Associations between screen time and lower psychological well-being among children and adolescents: Evidence from a population-based study. *Prev Med Reports*. 2018;12:271–283.

17. Maras D, Flament MF, Murray M, Buchholz A, Henderson KA, Obeid N, Goldfield GS. Screen time is associated with depression and anxiety in Canadian youth. *Prev Med (Baltim)* [Internet]. 2015;73:133–138.

18. Costigan SA, Barnett L, Plotnikoff RC, Lubans DR. The health indicators associated with screen-based sedentary behavior among adolescent girls: A systematic review [Internet]. J. Adolesc. Heal. 2013 [cited 2021 May 13];52:382–392. Available from: https://pubmed.ncbi.nlm.nih.gov/23299000/

19. Fitzgerald CT, Messias E, Buysse DJ. Teen Sleep and Suicidality: Results from the Youth Risk Behavior Surveys of 2007 and 2009. *J Clin Sleep Med* [Internet]. 2011;7:351–356. Available from: http://www.cdc.gov/HealthyYouth/yrbs/index.htm.

20. Matamura M, Tochigi M, Usami S, Yonehara H, Fukushima M, Nishida A, Togo F, Sasaki T. Associations between sleep habits and mental health status and suicidality in a longitudinal survey of monozygotic twin adolescents. *J Sleep Res* [Internet]. 2014 [cited 2021 May 13];23:292–296. Available from: https://onlinelibrary.wiley.com/doi/full/10.1111/jsr.12127

21. Sarris J, Logan AC, Akbaraly TN, Amminger GP, Balanzá-Martínez V, Freeman MP, Hibbeln J, Matsuoka Y, Mischoulon D, Mizoue T, Nanri A, Nishi D, Ramsey D, Rucklidge JJ, Sanchez-Villegas A, Scholey A, Su KP, Jacka FN. Nutritional medicine as mainstream in psychiatry [Internet]. The Lancet Psychiatry. 2015 [cited 2021 May 13];2:271–274. Available from: https://pubmed.ncbi.nlm.nih.gov/26359904/

22. Pischke CR, Scherwitz L, Weidner G, Ornish D. Long-Term Effects of Lifestyle Changes on Well-Being and Cardiac Variables Among Coronary Heart Disease Patients. *Heal Psychol* [Internet]. 2008 [cited 2021 May 13];27:584–592. Available from: /record/2008-13168-010

23. Laranjo L, Arguel A, Neves AL, Gallagher AM, Kaplan R, Mortimer N, Mendes GA, Lau AYS. The influence of social networking sites on health behavior change: a systematic review and meta-analysis. *J Am Med Informatics Assoc* [Internet]. 2015;22:243–256. Available from: https://academic.oup.com/jamia/article/22/1/243/833940

24. Larun L, Nordheim L V, Ekeland E, Hagen KB, Heian F. Exercise in prevention and treatment of anxiety and depression among children and young people (review). *Cochrane Database Syst Rev*. 2006;3:CD004691.

25. Ahn S, Fedewa AL. A Meta-analysis of the Relationship Between Children’s Physical Activity and Mental Health. *J Pediatr Psychol* [Internet]. 2011;36:385–397. Available from: https://academic.oup.com/jpepsy/article-lookup/doi/10.1093/jpepsy/jsq107

26. Babic MJ, Smith JJ, Morgan PJ, Eather N, Plotnikoff RC, Lubans DR. Longitudinal associations between changes in screen-time and mental health outcomes in adolescents. *Ment Health Phys Act* [Internet]. 2017;12:124–131. Available from: https://linkinghub.elsevier.com/retrieve/pii/S1755296616300862

27. Rollinson R, Price I, Gee B, Lyons J, Carroll B, Wilson J, Clarke T. Low-intensity sleep intervention in a youth mental health service: a case series analysis. *Behav Cogn Psychother* [Internet]. 2021;49:62–75. Available from: https://www.cambridge.org/core/product/identifier/S135246582000051X/type/journal_article

28. Larun L, Nordheim L V, Ekeland E, Hagen KBKB, Heian F. Exercise in prevention and treatment of anxiety and depression among children and young people. *Cochrane Database Syst Rev* [Internet]. 2006;3:CD004691. Available from: http://www.scopus.com/inward/record.url?eid=2-s2.0-33750093798&partnerID=40&md5=f968056774dab0f98c4bc909f56be1dd

29. Maniccia DM, Davison KK, Marshall SJ, Manganello JA, Dennison BA. A Meta-analysis of Interventions That Target Children’s Screen Time for Reduction. *Pediatrics* [Internet]. 2011;128:e193–e210. Available from: https://publications.aap.org/pediatrics/article/128/1/e193/30302/A-Meta-analysis-of-Interventions-That-Target

30. Freeman D, Sheaves B, Goodwin GM, Yu L-M, Nickless A, Harrison PJ, Emsley R, Luik AI, Foster RG, Wadekar V, Hinds C, Gumley A, Jones R, Lightman S, Jones S, Bentall R, Kinderman P, Rowse G, Brugha T, Blagrove M, Gregory AM, Fleming L, Walklet E, Glazebrook C, Davies EB, Hollis C, Haddock G, John B, Coulson M, Fowler D, Pugh K, Cape J, Moseley P, Brown G, Hughes C, Obonsawin M, Coker S, Watkins E, Schwannauer M, MacMahon K, Siriwardena AN, Espie CA. The effects of improving sleep on mental health (OASIS): a randomised controlled trial with mediation analysis. *The Lancet Psychiatry* [Internet]. 2017;4:749–758. Available from: https://linkinghub.elsevier.com/retrieve/pii/S2215036617303280

31. Roberts KC, Yao X, Carson V, Chaput JP, Janssen I, Tremblay MS. Meeting the Canadian 24-hour movement guidelines for children and youth. *Heal Reports* [Internet]. 2017;28:3–7.

32. Emmelkamp PM, David D, Beckers T, Muris P, Cuijpers P, Lutz W, Andersson G, Araya R, Banos Rivera RM, Barkham M, Berking M, Berger T, Botella C, Carlbring P, Colom F, Essau C, Hermans D, Hofmann SG, Knappe S, Ollendick TH, Raes F, Rief W, Riper H, Van Der Oord S, Vervliet B. Advancing psychotherapy and evidence-based psychological interventions. *Int J Methods Psychiatr Res*. 2014;23:58–91.

33. Kaess M, Ritter S, Lustig S, Bauer S, Becker K, Eschenbeck H, Moessner M, Rummel-Kluge C, Salize H-J, Thomasius R, Resch F, Koenig J. Promoting Help-seeking using E-technology for ADolescents with mental health problems: study protocol for a randomized controlled trial within the ProHEAD Consortium. *Trials* [Internet]. 2019;20:94. Available from: https://trialsjournal.biomedcentral.com/articles/10.1186/s13063-018-3157-7

34. Goodman R. Psychometric Properties of the Strengths and Difficulties Questionnaire. *J Am Acad Child Adolesc Psychiatry* [Internet]. 2001 [cited 2021 May 6];40:1337–1345. Available from: www.sdqinfo.com

35. University of Washington Seattle Quality of Life Group. Youth Quality of Life-short form instrument. 2016.

36. Canadian Society for Exercise Physiology. Physical Activity Training for Health (CSEP-PATH). Ottawa, Ontario: Canadian Society for Exercise Physiology; 2013.

37. Storfer-Isser A, Lebourgeois MK, Harsh J, Tompsett CJ, Redline S. Psychometric properties of the Adolescent Sleep Hygiene Scale. *J Sleep Res* [Internet]. 2013;22:707–716. Available from: http://doi.wiley.com/10.1111/jsr.12059

38. Hay JA, Cairney J. Development of the Habitual Activity Estimation Scale for clinical research: A systematic approach. *Pediatr Exerc Sci* [Internet]. 2006;18:193–202. Available from: https://www.scopus.com/inward/record.url?eid=2-s2.0-33646580393&partnerID=40&md5=678c0beb43ccf08f2a1209ee96be9d95

39. Hardy LL, Booth ML, Okely AD. The reliability of the Adolescent Sedentary Activity Questionnaire (ASAQ). *Prev Med (Baltim)* [Internet]. 2007;45:71–74. Available from: https://linkinghub.elsevier.com/retrieve/pii/S0091743507001430

40. Trost SG, Pate RR, Freedson PS, Sallis JF, Taylor WC. Using objective physical activity measures with youth: how many days of monitoring are needed? *Med Sci Sport Exerc* [Internet]. 2000;32:426–431. Available from: http://www.ncbi.nlm.nih.gov/entrez/query.fcgi?cmd=Retrieve&db=PubMed&dopt=Citation&list_uids=10694127

41. Trost SG, Loprinzi PD, Moore R, Pfeiffer KA. Comparison of accelerometer cut points for predicting activity intensity in youth. *Med Sci Sports Exerc* [Internet]. 2011;43:1360–8. Available from: http://www.ncbi.nlm.nih.gov/pubmed/21131873

42. Thai H, Davis CG, Stewart N, Gunnell KE, Goldfield GS. The Effects of Reducing Social Media Use on Body Esteem Among Transitional-Aged Youth. *J Soc Clin Psychol* [Internet]. 2021;40:481–507. Available from: https://guilfordjournals.com/doi/10.1521/jscp.2021.40.6.481

43. Mathai J, Anderson P, Bourne A. Use of the Strengths and Difficulties Questionnaire as an Outcome Measure in a Child and Adolescent Mental Health Service. *Australas Psychiatry* [Internet]. 2003;11:334–337. Available from: http://journals.sagepub.com/doi/10.1046/j.1440-1665.2003.00544.x

44. LeBlanc JM, Norris M, S. Lee J, Gray C, Cloutier P, Robb M, Longmuir PE. Lifestyle issues are a frequent component of children’s mental health treatment: Retrospective data from a pediatric teriary care center. *Manuscr Prog*. 2024;Manuscript.
