# Supplementary material for: Evaluation of the novel HEalthy Lifestyle Project (HELP) youth mental health e-intervention for lifestyle behaviour change and mental healthcare system impact: A randomized controlled trial protocol
Source: PLoS One. 2025 Nov 3;20(11):e0332363. doi: 10.1371/journal.pone.0332363 (PMC12582452; doi:10.1371/journal.pone.0332363)
Supplement: S3 File — HELP lifestyle interventions concept study. LeBlanc JM, Norris M, S. Lee J, Gray C, Cloutier P, Robb M, al. Lifestyle issues are a frequent component of children’s mental health treatment: Retrospective data from a pediatric tertiary care center. Data is available from the corresponding author on request. (DOCX) [file pone.0332363.s003.docx]

**Lifestyle Issues are a Frequent Component of Children’s Mental Health Treatment: Retrospective Data from a Pediatric Tertiary Care Center**

**Abstract:**

Problem: Healthy lifestyle behaviours promote mental well-being and benefit treatment. Allied health professionals could support positive lifestyles among youth receiving mental health services.

Methods: Random retrospective sample of 102 children/adolescents (51 female, 11.6±3.4 years). All first-year treatment visits were reviewed. Descriptive statistics summarized sleep, physical activity or screen time discussion/assessment or recommendation frequency. Age group/sex/referral reason were evaluated (chi-square).

Findings: Lifestyle was discussed with 95% of youth within 5.9±5.1 visits. Sleep was most frequent (83%), with sleep changes recommended for 46%. Physical activity assessment among 56% led to recommendations for 18%. Screen use changes recommended for 22%, with 62% assessed. Screen use discussions more likely among adolescents (p=0.02) and those with neurodevelopmental diagnoses (p=0.02).

Conclusions: Most youth receiving mental health treatment require lifestyle support. Systematic lifestyle screening and enhanced lifestyle behaviour change support by allied health professionals could decrease mental health service demands by enhancing youth mental well-being and facilitating treatment response.

Key words

child, adolescent, mental health services, lifestyle, counselling

Practitioner Points

Positive lifestyle behaviours contribute to mental well-being and can facilitate mental health treatment.

95% of youth receiving mental health treatment require lifestyle assessment or support.

Providing lifestyle assessment and support is an important role for nurses and other allied health professionals.

**Introduction**

Positive mental health is a vital component of overall wellbeing. In young people, positive mental health is associated with increased resilience (Mesman, Vreeker, & Hillegers, 2021), better self-esteem (Mann, 2004) and improved school performance (Malecki & Elliot, 2002) . Worldwide, 10-20% of children and youth are living with mental illness (Kieling et al., 2011). Even before the COVID-19 pandemic, rates of mental health care use among children and adolescents had increased in both acute care and primary care settings (Gandhi et al., 2016; Mapelli, Black, & Doan, 2015), with wait times for mental health assessments for children and youth exceeding 1 year in certain regions (Kowalewski, McLennan, & McGrath, 2011). Also of concern, less than one third of children and youth with symptoms of mental illness can access care from a mental health provider (Georgiades et al., 2019). Considering that up to 70% of mental illness has its onset in childhood or adolescence (Mood Disorders Society of Canada, 2006), efforts to optimize mental health during childhood and adolescence are especially important, as these are critical periods during which children and youth develop socio-emotional skills and behavioural patterns that will carry forward into adulthood. Furthermore, early intervention can prevent future adult mental health problems and has been shown to have significant net cost benefits (Kieling et al., 2011).

Engaging in regular physical activity, practicing good sleep hygiene, and using digital media sensibly are important lifestyle behaviours that contribute to positive mental health. Current guidelines for children and youth recommend at least 60 minutes of moderate-to-vigorous physical activity and several hours of light physical activity per day, 8-11 hours of uninterrupted sleep, and no more than 2 hours per day of recreational screen time (Roberts et al., 2017; World Health Organization, 2020). A 2017 survey of Canadians aged five to 16 years old found that 82.5% of children and youth do not meet these guidelines (Roberts et al., 2017). Poor sleep habits (Owens et al., 2014), increased duration of screen time (Maras et al., 2015), and low levels of physical activity (Cooney et al., 2013; Walsh, 2011) have each been associated with worse mental health outcomes, including the development of depression and anxiety (Suchert, Hanewinkel, & Isensee, 2015). Of importance, published evidence points to the benefits of lifestyle change on youth mental health symptoms (Walsh, 2011). Evidence suggests that exercise, defined as structured physical activity intended to increase fitness, can in certain circumstances be as effective as psychotherapy and medication for treating depressive disorders (Cooney et al., 2013). Modest extensions of sleep duration have been shown to significantly improve emotional regulation (Gruber, Cassoff, Frenette, Wiebe, & Carrier, 2012). Decreased sedentary behaviour and screen time are associated with a decreased risk of depression (Liu, Wu, & Yao, 2016). In keeping with these findings, a recent cross-sectional study of children completed during the COVID-19 pandemic revealed that increased physical activity and less screen time were associated with better mental health, even when accounting for pandemic stressors (Tandon, Zhou, Johnson, Gonzalez, & Kroshus, 2021).

While the link between healthy lifestyle behaviours and mental health is recognized, interventions to improve these behaviours have not been sufficiently studied in youth with mental distress (Walsh, 2011). Furthermore, it is unclear whether mental health care providers supporting children and adolescents routinely assess, engage in discussions about, or make recommendations to their patients about lifestyle behaviours. To better understand the need for, and current practices regarding lifestyle behaviour support in youth mental health practice, this study sought to understand how frequently sleep, physical activity or screen time were assessed, discussed or recommended as needing behaviour change during mental health consultations at a tertiary care pediatric hospital. We also sought to assess whether differences in age, sex or diagnosis were correlated with differences in the frequency of lifestyle behaviour discussions or recommendations. We hypothesized that all patients would be assessed for sleep, physical activity and screen time habits, with assessments occurring during the first two visits. We also hypothesized that discussions and recommendations for lifestyle behaviours would be documented as needed but that patients would not be referred for additional behaviour change support as such support is not routinely incorporated into the role of nurses or other allied health professionals practising in pediatric institutions.

**Methods**

***Target Population***

A retrospective chart review of health records was completed for children and adolescents assessed by a specialized outpatient mental health team at a tertiary care hospital between January 1st, 2017 and December 31st, 2017. Patients were referred by a family doctor or pediatrician, with referrals generally being for diagnostic clarification and/or mental health treatment recommendations. Patients were provided with support services tailored to individual mental health needs, with treatment duration and available programs being highly diverse. The study was approved by the Research Ethics Board of the treating institution. A waiver of informed consent allowing researchers to access medical chart data without patient/parent contact was issued by the Research Ethics Board due to the retrospective nature of the study.

The target sample was 100 patients randomly selected from 2017 initial assessment visits completed by all providers (i.e., psychiatrists, pediatricians, psychologists, and social workers). There were no exclusion criteria. The sampling strategy balanced age and biological sex: patients were categorized by sex (as indicated in the medical chart), and then ordered by age. Of note, only one chart indicated gender diversity (trans male). From the total number of available patients in both the male (n=277) and female (n=281) cohorts, every 6th patient was selected to provide a representative sample. Review and coding of the medical charts for all mental health sessions occurring within one year of each patient’s initial visit was completed.

***Data Extraction***

Two of the co-authors independently assessed charts using a standardized data abstraction form (see Supplementary file 1) that captured patient characteristics including age, sex, diagnoses, medications, type of health care provider and number of visits. In addition, information relating to the lifestyle behaviours under investigation (sleep, physical activity, or screen use) were collected. Documentation for each visit was categorized under the following headings for each lifestyle behaviour: (1) None: lifestyle behaviour was not charted; (2) Assessed: Lifestyle behaviour was assessed during visit 1 or 2; (3) Discussed: Lifestyle behaviour was raised with patient during a treatment visit and relevant findings documented, but no recommendations were charted; or (4) Recommendation: Lifestyle behaviour change was recommended to the patient by the provider and documented in the chart. To ensure we captured recommendations from the initial assessment, data from the first and second mental health visits were combined as assessment recommendations. Treatment visits were identified as the third or more visit. As a means of optimizing inter-rater agreement, a training sample of 10% of patients were reviewed by both authors coding the results, with the results compared and discrepancies discussed until consensus was achieved.

***Data Analyses***

Frequencies of discussed and recommended lifestyle behaviour changes were totaled for a) assessment visits (Visit 1 & 2) and b) treatment visits (Visit ≥ 3). The sample was divided into two age groups: children aged 4-12 years and adolescents aged 13-17 years. One-way ANOVA statistics were used to evaluate the impacts of age, sex, gender and type of diagnosis (anxiety, attention, neurodevelopmental, depression, disruptive, other) on lifestyle behaviour discussions or recommendations. Statistical significance was defined as p-values less than 0.05. Statistical analyses were performed using SPSS version 28 statistical software (IBM Corp., New York, USA 2021).

**Results**

***Participants***

The sample consisted of 51 female and 51 male patients (see Table 1, 51 boys, 50 girls, 1 trans boy). Mean age of the total sample was 11.6 ± 3.4 years (range 4 to 16). Including assessment and follow-up duration of 1 year, participants completed 601 outpatient visits with mental health professionals, half of which were with a psychiatrist (Table 1). The mean number of visits per participant was 5.9 ± 5.1.

***Lifestyle Behaviour Discussions and Recommendations***

The lifestyle behaviour most frequently addressed during mental health visits was sleep (Table 2). Across all visits, sleep was discussed with 85 patients (83%). Those discussions occurred with 78% of patients during assessment visits and with 47% of patients during treatment visits. Recommendations for changing sleep behaviours were provided to 39 youth (46% of those assessed), with recommendations occurring equally during assessment or treatment visits. The likelihood that sleep would be assessed (r=0.21, p<0.03) or sleep recommendations given (r=0.23, p=0.02) increased with the number of visits.

Across all visits, a discussion of screen use was noted in 63 (62%) patient charts, with 14 (22% of those assessed) receiving a recommendation to change their screen behaviour. Screen use was discussed, or recommendations made only during treatment visits among adolescent boys. The frequency of screen time recommendations also increased with the number of visits (r=0.28, p=0.005).

Although physical activity was discussed with a total of 57 (56%) patients across all visits, only 10 (18% of those assessed) received a recommendation to change their physical activity behaviour. Physical activity discussions occurred more frequently during assessment visits with younger children. Physical activity was more likely to be assessed as the number of visits increased (r=0.23, p=002).

Considering all visits in all patients over one year of follow up, at least one lifestyle behaviour was addressed at least once with 95% of the patients (84% sleep; 64% screen time; 57% physical activity). Only 38 patients (37%) had discussions or recommendations for all three lifestyle behaviours noted in their medical chart.

***Impact of Age, Sex and Diagnosis on Lifestyle Behaviours***

Overall, the proportion of patients for whom any lifestyle behaviour was discussed or recommended did not differ by sex or gender. Physical activity was most likely to be discussed with children during assessment visits (p=0.05) and with adolescents during treatment visits (p=0.02). During treatment visits, screen use tended to be assessed more often (p=0.07) and recommendations for changing behaviour made (p=0.02) among adolescents. Screen use was also more likely to be assessed among youth with neurodevelopmental diagnoses (p=0.02 and p=0.04 for assessment and treatment visits, respectively). Sleep recommendations were less likely among youth with a disruptive diagnosis (p=0.01).

**Discussion**

To our knowledge, previous studies have not evaluated the rate at which mental health providers assess, discuss or recommend lifestyle behaviour changes to their pediatric patients. Despite recommendations and endorsements by national pediatric specialty societies (Canadian Paediatric Society Digital Health Task Force, 2019; Gruber et al., 2014; Hill et al., 2016) regarding the importance of these behaviours, we found that all three lifestyle behaviours were assessed or discussed with only 38 of 102 patients (37%). Although providers did consistently (95%) mention in the chart at least one lifestyle behaviour per patient, a standardized approach to the assessment of all three lifestyle behaviour factors was not routinely observed. The assessment of at least one lifestyle behaviour occurred in 95 (93%) patients with almost half (46%) receiving at least one recommendation about a lifestyle change. While speculative, the lack of a standardized instrument/approach to lifestyle behaviour assessment or a lack of specialized knowledge regarding lifestyle behaviour screening and recommendations may have contributed to the limited discussions recorded in the patient chart. Future research should evaluate the utility of a standardized instrument to screen patients for specific lifestyle behaviours, the role of nurses and other allied health professionals in assessing lifestyle behaviours and the impact of lifestyle specialist support for increasing overall uptake and application of the behaviour change recommendations.

Of the three lifestyle behaviour categories, discussions of sleep were noted most frequently. Almost half (46%) of the patients whose sleep habits were assessed were provided with a recommendation for sleep behaviour change. Without a standardized mechanism of reporting and documentation, it is difficult to delineate to what extent these values represent appropriate or missed opportunities for supportive counselling. Although the quality and types of questions relating to each of the lifestyle behaviour categories was not studied, duration and quality of sleep are important aspects of overall health for all pediatric patients, regardless of the presenting mental health complaint. With that in mind, it stands to reason that a majority of patients assessed for mental health concerns could potentially benefit from a standardized assessment of sleep (e.g., Adolescent Sleep Hygiene Scale (Storfer-Isser *et al.*, 2013)), which could then help to inform counselling over the course of treatment.

Multiple studies have suggested that rates of sedentary behaviour and screen use are unacceptably high among youth and are linked to poor mental health (Bang, Roberts, Chaput, Goldfield, & Prince, 2020; Gunnell et al., 2016). In this study, just over half of the patients were assessed for their physical activity (56%) or screen time (62%) behaviours. Pre-pandemic, 20% of students in grades 7-12 spent 5 hours or more per day on social media, up from 11% in 2013 (Paglia-Boak et al., 2015). Average daily screen time among this age group has been cited at 7.8 h per day (Leatherdale & Ahmed, 2011). Studies also suggest that only one third of children and youth meet the recommended 60 minutes of moderate-to-vigorous physical activity per day (Roberts et al., 2017). Among children and adolescents, increasing moderate-to-vigorous physical activity and limiting sedentary time are beneficial for mental health (Roberts et al., 2017; Trinh, Wong, & Faulkner, 2015), with excessive screen/social media use being associated with depressive symptoms (Canadian Paediatric Society Digital Health Task Force, 2019). Given the prevalence of inadequate physical activity and excessive screen use and their link to mental health, enabling nurses and other allied health professionals to screen for these concerns among all pediatric patients and refer at risk patients for specialist lifestyle support is recommended. Incorporating a standardized tool that ensures that screen time and physical activity are discussed with patients within the assessment period (visits 1 and 2) would ensure that mental health providers are aware of important modifiable risk factors applicable to the majority of children and youth.

Evidence is still emerging for a causal link between lifestyle behaviours and mental health benefits (Biddle, Ciaccioni, Thomas, & Vergeer, 2019), but the positive correlation between healthy lifestyle behaviours and positive mental well-being is well-documented in the literature. A meta-analyses of studies evaluating the effects of physical activity on pediatric mental health showed significant positive effects on mental health outcomes (Ahn & Fedewa, 2011). Interestingly, recent research has found that in adolescents, adhering to multiple lifestyle recommendations (specifically related to diet, physical activity, sedentary behaviour and sleep) has a positive cumulative effect, where adherence to more lifestyle behaviours is associated with fewer mental health visits (Loewen et al., 2019). Given the current lifestyle behaviours of children and adolescents, early targeted screening and recommendations relating to lifestyle behaviour modification with referral for lifestyle specialist support may help to relieve some of the current burden on mental health services. Lifestyle behaviour change strategies are accessible to the majority of patients and risk of harm is very low (Walsh, 2011). Although discussions regarding the importance of lifestyle behaviours does not require extensive training or time to implement, referral of patients to specialist support is recommended if lifestyle behaviour change is required. Changing lifestyle behaviours is difficult and involves more than advice to “eat healthier” or “be more active”. Clinicians who have advanced knowledge of the wide variety of lifestyle behaviour options are better positioned to provide patients with the personalized, on-going support needed to achieve long-term behaviour change.

Our study is not without limitations. Data were collected retrospectively. In addition to general considerations relating to documentation, it is possible that specific lifestyle behaviour documentation was not included in notes and summaries as it was felt to not substantially contribute to the overall case history. As the health record is intended to reflect the essential elements of an encounter, we would suggest that lifestyle behaviour topics (if indeed discussed) would have warranted inclusion in transcribed notes. Furthermore, if a provider does not chart recommendations that they have made to the patient, primary health care providers would be unable to support lifestyle behaviour recommendations during their own follow-up visits. We also recognize that the likelihood of charting lifestyle discussions or asking about certain lifestyle behaviours may be specific to certain providers and/or disciplines.

**Conclusions**

Overall, this study found varying rates of screening and low overall rates of lifestyle behaviour recommendations among patients receiving mental health treatment. Screen use was more likely to be addressed with adolescents and among youth with neurodevelopmental diagnoses. Otherwise, sex, gender, age or reason for referral were not related to the frequency of lifestyle discussions or recommendations. Although speculative, it may be that the overwhelming demand for mental health support in youth may limit the ability of mental health professionals to assess lifestyle behaviours. Rather, their time is focused on delivering the mental health treatments for which they are trained. Nevertheless, the links between lifestyle behaviours and youth mental well-being are clear. Given the need to systematically assess lifestyle behaviours and the frequency of behaviour change recommendations identified in this study (10% to 25% of patients with only 50% to 85% of patients assessed), incorporating lifestyle assessment within the roles of nurses and other allied health professionals is recommended. Specialist support for lifestyle behaviour change within mental health services would enhance the mental well-being of patients and optimize their readiness to benefit from mental health treatment. The availability of a lifestyle behaviour change specialist would also enable referral for specific lifestyle supports as needed, enhancing the support time provided to patients and enabling mental health professionals to focus on psychosocial treatment strategies. Other possible explanations for our findings should also be investigated. It is possible that a proportion of the providers completing mental health assessments were not aware of current evidence regarding the links between lifestyle behaviour and mental health. Providers may also be hesitant to raise topics and identify concerns if they are not confident to engage patients on these topics or are not aware of avenues for providing additional lifestyle support. It is also possible that providers undertook these discussions but did not document these parts of their assessments if the findings were of relatively less concern. Finally, it may be that mental health providers were focused primarily on delivering the psychosocial treatment strategies for which they have been trained.

Future research is recommended to build upon initial results obtained herein and to directly investigate the quality of questions used for assessment and counselling purposes and the role of nurses and allied health professionals in conducting these assessments. A standardized instrument that assessed (and ideally tracked) lifestyle behaviour-related endeavors would enable real-time feedback regarding compliance with any stated lifestyle behaviour recommendations. Prospective studies could also assess how counselling interventions and communication styles with patients impact lifestyle behaviour change or influence overall mental health outcomes.

Given the increased demand for mental health assessment and intervention, and the limited resources available, it is critical that the expertise of all health care providers be utilized to address modifiable lifestyle risk factors at the time of first mental health assessment. In this study, while 95% of patients were asked about at least one lifestyle behaviour over all encounters, we demonstrated low overall rates of discussion across the combined categories of sleep, physical activity, and screen use. Although screening for and subsequently making lifestyle behaviour recommendations to patients does not guarantee implementation or subsequent derived benefit, education and discussion around these themes are steps towards the encouragement of lifestyle changes that support positive mental health and may reduce the risk for mental health issues among children and adolescents who are at risk.

**Clinical Significance**

Research clearly links lifestyle behaviours (i.e., sleep, physical activity, screen time) with youth mental well-being but the assessment of these behaviours by mental health professionals is inconsistent, suggesting an important role for nurses and other allied health professionals. Recommendations for lifestyle behaviour change were provided to one fifth (screen time 22%; physical activity 18%) to one half (sleep 46%) of patients assessed, representing a substantial need for lifestyle behaviour change specialist support. Engaging lifestyle specialists to support behavior change may enhance mental well-being and reduce workload/wait times for mental health specialists.

**References**

Ahn, S., & Fedewa, A. L. (2011). A Meta-analysis of the Relationship Between Children’s Physical Activity and Mental Health. *Journal of Pediatric Psychology*, *36*(4), 385–397. https://doi.org/10.1093/jpepsy/jsq107

Bang, F., Roberts, K. C., Chaput, J.-P., Goldfield, G. S., & Prince, S. A. (2020). Physical activity, screen time and sleep duration: Combined associations with psychosocial health among Canadian children and youth. *Health Reports / Statistics Canada, Canadian Centre for Health Information*, *31*(5), 9–16.

Biddle, S. J. H., Ciaccioni, S., Thomas, G., & Vergeer, I. (2019). Physical activity and mental health in children and adolescents: An updated review of reviews and an analysis of causality. *Psychology of Sport and Exercise*, *42*, 146–155. https://doi.org/10.1016/j.psychsport.2018.08.011

Canadian Paediatric Society Digital Health Task Force. (2019). Digital media: Promoting healthy screen use in school-aged children and adolescents. *Paediatrics & Child Health*, *24*(6), 402–408. https://doi.org/10.1093/pch/pxz095

Cooney, G. M., Dwan, K., Greig, C. A., Lawlor, D. A., Rimer, J., Waugh, F. R., … Mead, G. E. (2013). Exercise for depression. *Cochrane Database of Systematic Reviews*, (9). https://doi.org/10.1002/14651858.CD004366.pub6

Gandhi, S., Chiu, M., Lam, K., Cairney, J. C., Guttmann, A., & Kurdyak, P. (2016). Mental Health Service Use Among Children and Youth in Ontario: Population-Based Trends Over Time. *Canadian Journal of Psychiatry. Revue Canadienne de Psychiatrie*, *61*(2), 119–124. https://doi.org/10.1177/0706743715621254

Georgiades, K., Duncan, L., Wang, L., Comeau, J., Boyle, M. H., & 2014 Ontario Child Health Study Team. (2019). Six-Month Prevalence of Mental Disorders and Service Contacts among Children and Youth in Ontario: Evidence from the 2014 Ontario Child Health Study. *Canadian Journal of Psychiatry. Revue Canadienne de Psychiatrie*, *64*(4), 246–255. https://doi.org/10.1177/0706743719830024

Gruber, R., Carrey, N., Weiss, S. K., Frappier, J. Y., Rourke, L., Brouillette, R. T., & Wise, M. S. (2014). Position statement on pediatric sleep for psychiatrists. *Journal of the Canadian Academy of Child and Adolescent Psychiatry = Journal de l’Academie Canadienne de Psychiatrie de l’enfant et de l’adolescent*, *23*(3), 174–195. Retrieved from http://www.ncbi.nlm.nih.gov/pubmed/25320611

Gruber, R., Cassoff, J., Frenette, S., Wiebe, S., & Carrier, J. (2012). Impact of Sleep Extension and Restriction on Children’s Emotional Lability and Impulsivity. *Pediatrics*, *130*(5), e1155–e1161. https://doi.org/10.1542/peds.2012-0564

Gunnell, K. E., Flament, M. F., Buchholz, A., Henderson, K. A., Obeid, N., Schubert, N., & Goldfield, G. S. (2016). Examining the bidirectional relationship between physical activity, screen time, and symptoms of anxiety and depression over time during adolescence. *Preventive Medicine*, *88*, 147–152. https://doi.org/10.1016/j.ypmed.2016.04.002

Hill, D., Ameenuddin, N., Reid Chassiakos, Y. (Linda), Cross, C., Radesky, J., Hutchinson, J., … Swanson, W. S. (2016). Media Use in School-Aged Children and Adolescents. *Pediatrics*, *138*(5), e20162592. https://doi.org/10.1542/peds.2016-2592

Kieling, C., Baker-Henningham, H., Belfer, M., Conti, G., Ertem, I., Omigbodun, O., … Rahman, A. (2011). Child and adolescent mental health worldwide: evidence for action. *The Lancet*, *378*(9801), 1515–1525. https://doi.org/10.1016/S0140-6736(11)60827-1

Kowalewski, K., McLennan, J. D., & McGrath, P. J. (2011). A preliminary investigation of wait times for child and adolescent mental health services in Canada. *Journal of the Canadian Academy of Child and Adolescent Psychiatry = Journal de l’Academie Canadienne de Psychiatrie de l’enfant et de l’adolescent*, *20*(2), 112–119. Retrieved from http://www.ncbi.nlm.nih.gov/pubmed/21541100

Leatherdale, S. T., & Ahmed, R. (2011). Screen-based sedentary behaviours among a nationally representative sample of youth: Are Canadian kids couch potatoes? *Chronic Diseases and Injuries in Canada*, *31*(4), 141–146. https://doi.org/10.1037/e528202014-001

Liu, M., Wu, L., & Yao, S. (2016). Dose-response association of screen time-based sedentary behaviour in children and adolescents and depression: a meta-analysis of observational studies. *British Journal of Sports Medicine*, *50*(20), 1252–1258. Retrieved from file:///V:/HALO/HALO Staff/Longmuir/New Literature B/Liu.ST.Depression.Meta-analysis.pdf

Loewen, O. K., Maximova, K., Ekwaru, J. P., Faught, E. L., Asbridge, M., Ohinmaa, A., & Veugelers, P. J. (2019). Lifestyle Behavior and Mental Health in Early Adolescence. *Pediatrics*, *143*(5), e20183307. https://doi.org/10.1542/peds.2018-3307

Malecki, C. K., & Elliot, S. N. (2002). Children’s social behaviors as predictors of academic achievement: A longitudinal analysis. *School Psychology Quarterly*, *17*(1), 1–23. https://doi.org/10.1521/scpq.17.1.1.19902

Mann, M. (2004). Self-esteem in a broad-spectrum approach for mental health promotion. *Health Education Research*, *19*(4), 357–372. https://doi.org/10.1093/her/cyg041

Mapelli, E., Black, T., & Doan, Q. (2015). Trends in Pediatric Emergency Department Utilization for Mental Health-Related Visits. *The Journal of Pediatrics*, *167*(4), 905–910. https://doi.org/10.1016/j.jpeds.2015.07.004

Maras, D., Flament, M. F., Murray, M., Buchholz, A., Henderson, K. A., Obeid, N., & Goldfield, G. S. (2015). Screen time is associated with depression and anxiety in Canadian youth. *Preventive Medicine*, *73*, 133–138. Retrieved from file:///V:/HALO/HALO Staff/Longmuir/New Literature B/Screentime.Depression.anxiety.PrevMed.Feb.2015.pdf

Mental Health Commission of Canada. (2013). *Making the Case for Investing in Mental Health in Canada*. Retrieved from https://books-scholarsportal-info.proxy.bib.uottawa.ca/en/read?id=/ebooks/ebooks0/gibson_cppc-chrc/2013-04-25/1/10666713

Mesman, E., Vreeker, A., & Hillegers, M. (2021). Resilience and mental health in children and adolescents: an update of the recent literature and future directions. *Current Opinion in Psychiatry*, *34*(6), 586–592. https://doi.org/10.1097/YCO.0000000000000741

Mood Disorders Society of Canada. (2006). *The Human Face of Mental Health and Mental Illness in Canada*. Retrieved from https://www.phac-aspc.gc.ca/publicat/human-humain06/pdf/human_face_e.pdf

Owens, J., Au, R., Carskadon, M., Millman, R., Wolfson, A., Braverman, P. K., … O’Brien, R. F. (2014). Insufficient sleep in adolescents and young adults: An update on causes and consequences. *Pediatrics*, *134*(3), e921–e932. https://doi.org/10.1542/peds.2014-1696

Paglia-Boak, A., Hamilton, H. A., Adlaf, E. M., Beitchman, J., Wolfe, D., & Mann, R. E. (2015). *The mental health and well-being of Ontario students 1991-2013: Detailed OSDUHS findings*. https://doi.org/10.1037/e528202014-001

Roberts, K. C., Yao, X., Carson, V., Chaput, J. P., Janssen, I., & Tremblay, M. S. (2017). Meeting the Canadian 24-hour movement guidelines for children and youth. *Health Reports*, *28*(10), 3–7. Retrieved from file:///V:/HALO/HALO Staff/Longmuir/New Literature B/Meeting 24-hour guidlines for children and youth CHMS.pdf

Storfer-Isser, A., Lebourgeois, M. K., Harsh, J., Tompsett, C. J., & Redline, S. (2013). Psychometric properties of the Adolescent Sleep Hygiene Scale. *Journal of Sleep Research*, *22*(6), 707–716. https://doi.org/10.1111/jsr.12059

Suchert, V., Hanewinkel, R., & Isensee, B. (2015). Sedentary behavior and indicators of mental health in school-aged children and adolescents: A systematic review. *Preventive Medicine*, *76*, 48–57. https://doi.org/10.1016/j.ypmed.2015.03.026

Tandon, P. S., Zhou, C., Johnson, A. M., Gonzalez, E. S., & Kroshus, E. (2021). Association of Children’s Physical Activity and Screen Time With Mental Health During the COVID-19 Pandemic. *JAMA Network Open*, *4*(10), e2127892. https://doi.org/10.1001/jamanetworkopen.2021.27892

Trinh, L., Wong, B., & Faulkner, G. E. (2015). The Independent and Interactive Associations of Screen Time and Physical Activity on Mental Health, School Connectedness and Academic Achievement among a Population-Based Sample of Youth. *Journal of the Canadian Academy of Child and Adolescent Psychiatry = Journal de l’Academie Canadienne de Psychiatrie de l’enfant et de l’adolescent*, *24*(1), 17–24. Retrieved from http://www.ncbi.nlm.nih.gov/pubmed/26336376

Walsh, R. (2011). Lifestyle and mental health. *American Psychologist*, *66*(7), 579–592. https://doi.org/10.1037/a0021769

World Health Organization. (2020). WHO Guidelines on physical activity and sedentary behaviour: At a glance. In *World Health Organization*. Retrieved from https://apps.who.int/iris/bitstream/handle/10665/337001/9789240014886-eng.pdf?sequence=1&isAllowed=y
