# Supplementary material for: Evaluation of the novel HEalthy Lifestyle Project (HELP) youth mental health e-intervention for lifestyle behaviour change and mental healthcare system impact: A randomized controlled trial protocol
Source: PLoS One. 2025 Nov 3;20(11):e0332363. doi: 10.1371/journal.pone.0332363 (PMC12582452; doi:10.1371/journal.pone.0332363)
Supplement: S4 File — (PDF) [file pone.0332363.s004.pdf]

Learn My Habits

Good mental health is being the best version of you in the moment!

Good mental health means that you can do things like:
* Handle the stresses in your day-to-day life,
* Get tasks and chores done,
* Connect positively with family and friends.

When you have concerns about your mental health it can be hard to make your bed, do your homework or see your friends.

If you struggle with your mental health, you are not alone!

There are 1.2 million Canadian youth who struggle with mental illness

Learn More

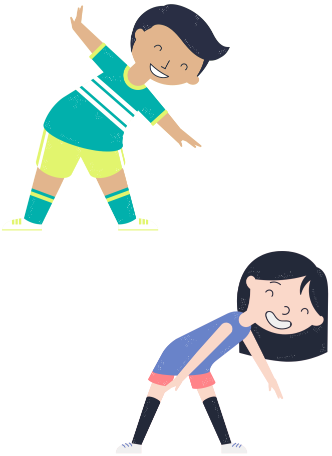

Being Active

It seems like you spend most of your time doing things that are *somewhat inactive*, such as reading, watching TV, playing video games, or being on the computer. Being somewhat inactive, rather than inactive, helps to improve your mental and emotional health. Congratulations - You have taken the first step and are on your way to becoming more active! Learn about small changes you can make to move from *somewhat inactive* to

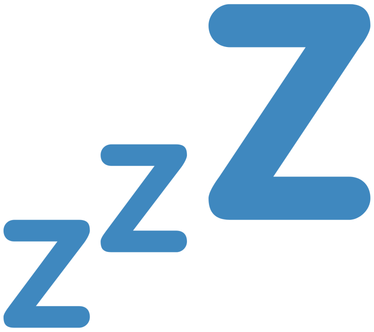

Sleep

You are currently "excelling" in your sleep habits! Awesome job, you have great sleep habits that support your mental and emotional health. It is great that you are ready to make changes to your behaviour. Check out the Plan sections for screen time and physical activity for easy and quick ways to get started, help with setting SMART goals, and more.

somewhat active or consider the pro's and con's of making that change.

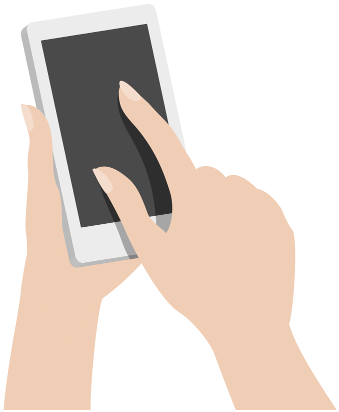

Screen Time

You are currently “Progressing” on your journey to a lifestyle with the screen time associated with positive mental and emotional health! Good job, you are off to a good start. Let’s continue the journey! Learn more about the benefits of reducing the time you spend on screens or consider the pro's and con's of reducing your screen time.

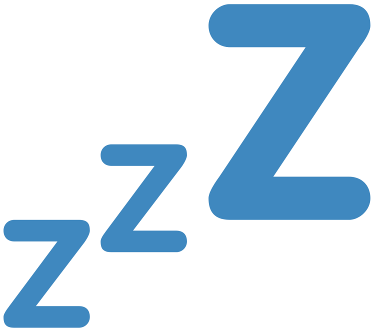

Life Balance

0

If you have questions, are experiencing difficulties, or need help with your behaviour change, please contact the kinesiologist using the contact information below:

Phone & E-mail

Phone: 613-737-7600 x 4003
Fax: 613-738-3908
cheoactive@cheo.on.ca

Business Hours

Monday – Friday
8.00 am – 4.00 pm
Weekend Closed

About Being Active

Background Information

Even just a little bit of physical activity can do so much good for you and your body! Physical activity can help you sleep better, it can help you have fun and connect with your friends and your family, and it can be something fun to look forward to in your day! Physical activity is related to good mental health and can reduce the symptoms related to mental illness. Physical activity can also help with reducing stress levels!

Learn

Let's Learn about Physical Activity!

Have you thought about making a change to your physical activity habits but want to learn more about physical activity and lifestyle first? See below to learn more!

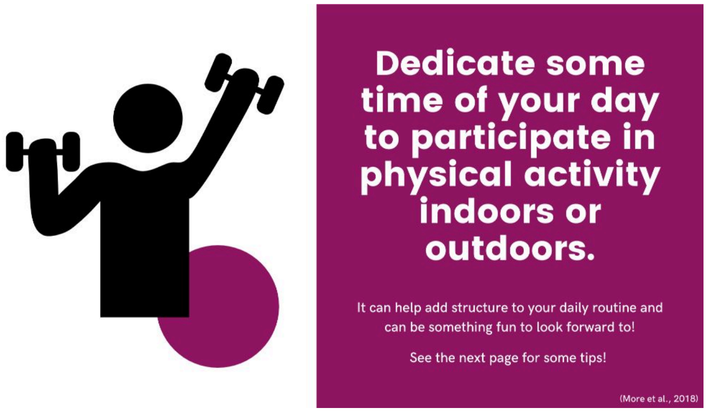

Pros & Cons Activity

Pros & Cons Activity

Plan

If you know what behaviour you are wanting to change for physical activity, click on the **goals** page, where we will provide you with a list of physical activity goals to work on!

Are you thinking of making a change towards your physical activity habits? Great! See below for some **small changes** you can start making to change your physical activity habits!

Small Changes to Help You Start Being Active

This resource has hundreds of fun activities and games.

Check out out CHEOActive website

While you are completing your plans, at any point if you feel you need to slow down and reflect on your body's cues, see the **Traffic Light exercise** below. It will help you slow down, feel comfortable and relaxed!

Traffic Light Exercise

Are you experiencing some roadblocks while completing the steps in your physical activity plans? That's okay! We are here to help! See the **"Dealing with Roadblocks"** button below to find some common roadblocks you may be experiencing, and some helpful tips on how you can overcome them!

Dealing with Roadblocks

More Links & Downloads

See below for some more fun links and downloads you can explore to help change your physical activity habits!

Title: 23 and ½ Hours: What Is The Single Best Thing We Can do for our Health?

Description: This video is a 10 minute educational video about physical activity and its positive benefits
Length: 10 minutes
Ages: 10 – 18+

Title: Follow Along with this Disney at Home Dance Workout!
Description: This video is a 20-minute cardio dance workout to Disney Songs.
Length: 20 minutes
Ages: 12 – 18+

Title: Follow Along with this Easy 10-Minute Morning "Wake Up" Workout
Description: A 10-minute standing morning workout video for beginners.
Length: 10 minutes
Ages: 10 – 18+

Title: Follow Along with this 15-Minute Full Body Stretch
Description: This video is a 15-minute full body stretch for beginners. It helps support mobility, flexibility, and relaxation.
Length: 15 minutes
Ages: 10 – 18+

Title: Follow Along with this Fun 13-Minute Family Cardio Workout
Description: Low impact cardio workout that is ten minutes long and requires no equipment
Length: 13 Minutes
Ages: 8-18 and parents

Title: Follow Along with these Soccer Dribbling Skills for Beginners
Description: This video is a tutorial on beginner-level soccer dribbling skills.
Ages: 10 – 18+

Title: Let's Learn some Martial Arts!
Description: This is a 'Guitar-Hero' or 'Dance-Dance Revolution' inspired video for physical activity – specifically martial arts! This interactive video teaches viewers martial arts basics in a fun way. Follow along with your hands and feet to the actions on the screen!
Length: 7 minutes
Ages: 10 – 18+

If you have questions, are experiencing difficulties, or need help with your behaviour change, please contact the kinesiologist using the contact information below:

Phone & E-mail

Business Hours

Phone: 613-737-7600 x 4003
Fax: 613-738-3908
cheoactive@cheo.on.ca

Monday – Friday
8.00 am – 4.00 pm
Weekend Closed

About Screen Time

Background Information

Leisure screen time can include activities like playing video games, watching a TV show, browsing on social media with a tablet (not including schoolwork!). Long periods of time spent staring at a screen can have a poor impact on your mental health. Even taking small breaks to break-up your screen time can be a big help on your health. For example, getting up and stretching your legs in between video games, or going for a short walk to the end of your street in between Netflix episodes. Reducing screen time doesn't mean you have to stop watching your favorite shows or talking to your friends on social media! Instead, try and limit these recreational screen time activities to 2 hours per day. This will do wonders for your body and your mental health AND will leave you with more free time to do other fun activities!

Learn

Have you thought about making a change to your screen time habits but want to learn more about screen time and lifestyle first? See below to learn more!

Case File #1: Screen Time

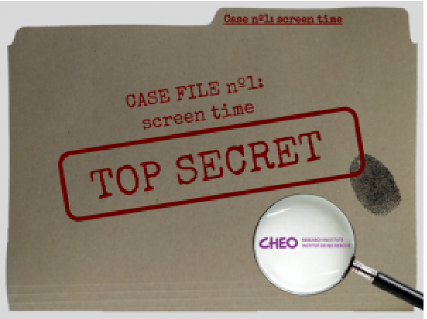

Pros & Cons Activity

Pros & Cons Activity

Plan

If you know what behaviour you are wanting to change for screen time, click on the **goals** page, where we will provide you with a list of screen time goals to work on!

Are you thinking of making a change towards your screen time habits? Great! See below for some **small changes** you can start making to change your screen time habits!

Small Changes Towards Reducing your Screen Time

While you are completing your plans, at any point if you feel you need to slow down and reflect on your body's cues, see the **Traffic Light exercise** below. It will help you slow down, feel comfortable and relaxed!

Traffic Light Exercise

Are you experiencing some roadblocks while completing the steps in your screen time plans? That's okay! We are here to help! See the **"Dealing with Roadblocks"** button below to find some common roadblocks you may be experiencing, and some helpful tips on how you can overcome them!

Dealing with Roadblocks

More Links & Downloads

See below for some more fun links and downloads you can explore to help change your screen time habits!

Title: Let's have a Yoga Break!
Description: This video is an 'office break' yoga practice routine. There are steps and movements throughout that can help reduce sedentary and can be done throughout the day.
Length: 14 minutes
Ages: 12 – 18+

Title: Click Here to Learn about Why Sitting is bad for you
Description: This video is an animation that describes the risks of sedentary behaviour and why it's important to be physically active when possible.
Length: 5 minutes
Ages: 10 – 18+

Title: Download this Habit Tracker App called "Flora" on your smart phone
Description: "Flora has you plant a virtual tree (alone or with friends) every time you set your 25-minute timer. If anyone who planted the tree touches their phone, the tree is killed. You also have the option to join the Flora Care service, which plants real trees when you reach your goal of total number of focused hours." (Popsugar)
Length: 25 minutes (or longer)
Ages: all ages

If you have questions, are experiencing difficulties, or need help with your behaviour change, please contact the kinesiologist using the contact information below:

Phone & E-mail

Phone: 613-737-7600 x 4003
Fax: 613-738-3908
cheoactive@cheo.on.ca

Business Hours

Monday – Friday
8.00 am – 4.00 pm
Weekend Closed

About Sleep

Background Information

Let's take a look at the impact that sleep can have on mental health. If you are between 5-13 years old, the recommended sleep time is 9-11 hours per night. If you are between 14-17 years old, the recommendation is 8-10 hours of sleep per night. Getting enough sleep has been linked with better mental health, like decreased anxiety, stress, and depression, lower risk of suicidal ideation and suicide, and better self-esteem.

Learn

Have you thought about making a change to your sleep habits but want to learn more about sleep and lifestyle first? See below to learn more!

Learn About Sleep With Us

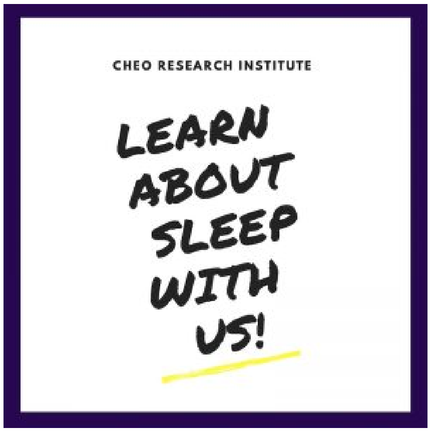

Did you know?

Plan

If you know what behaviour you are wanting to change for sleep, click on the **goals** page, where we will provide you with a list of sleep goals to work on!

Are you thinking of making a change towards your sleep habits? Great! See below for some **small changes** you can start making in your life to change your sleep habits!

Small Changes to Help You Get a Better Sleep

While you are completing your plans, at any point if you feel you need to slow down and reflect on your body's cues, see the **Traffic Light exercise** below. It will help you slow down, feel comfortable and relaxed!

Traffic Light Exercise

Are you experiencing some roadblocks while completing the steps in your sleep plans? That's okay! We are here to help! See the **"Dealing with Roadblocks"** button below to find some common roadblocks you may be experiencing, and some helpful tips on how you can overcome them!

Dealing with Roadblocks

More Links & Downloads

See below for some more fun links and downloads you can explore to help change your sleep habits!

Title: Follow Along with this 10 Minute Meditation to Help You Sleep
Description: This video is a 10-minute long guided meditation for sleep.
Length: 10 minutes
Ages: 10 – 18+

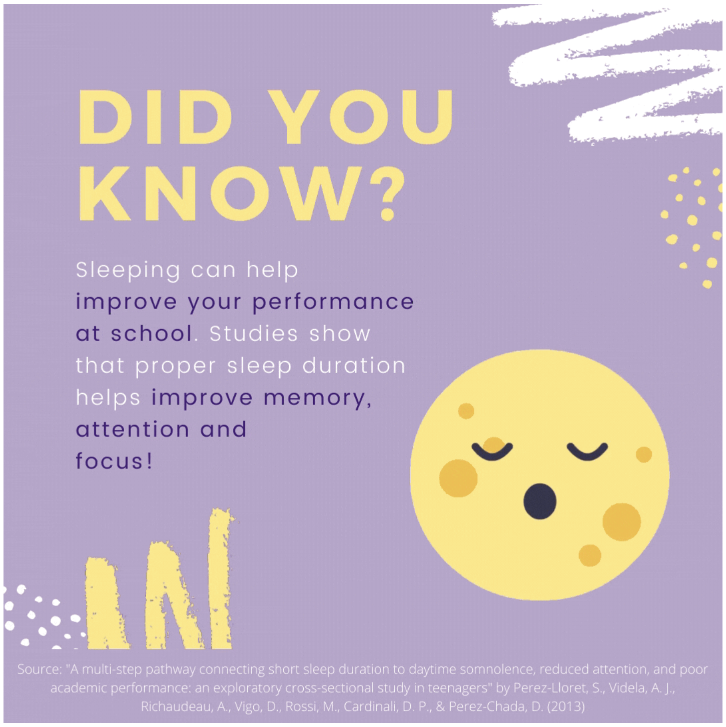

Creating a Bed Time Routine

0:00 / 0:41

What Makes Us Sleepy

0:00 / 1:25

Pros & Cons Activity

Pros & Cons Activity

Title: Follow Along with this 10-Minutes Meditation to help you Prepare to Sleep!
Description: This video is a 10-minute long guided meditation to prepare someone for sleep.
Length: 10 minutes
Ages: 10 – 18+

Title: Follow Along with this Yoga Mediation for some Stretching Before Bed!
Description: This video is 12-minute long guided yoga meditation that aims to help individual's stretch and release tension before going to bed.
Length: 12 minutes
Ages: 10 – 18+

Title: Follow Along with this Sleep Diary to Track your Sleep Progress!
Description: This is a daily sleep diary that aims to help improve sleep hygiene. Individuals can fill out the template and see their progress.
Length: 2 – 5 minutes
Ages: 8 – 18+

Title: Listen to this White Noise Video to Help You Fall Asleep and Stay Asleep
Description: This is a 10-hour white noise video (audio only) to improve sleep length and quality.
Length: 10 hours
Ages: any age

Title: Listen to this Brown Noise Video to Help You Fall Asleep and Stay Asleep
Description: This is an 8-hour long brown noise video (audio only) to improve sleep quality and quantity
Length: 8 hours
Ages: any age

Title: Listen to this video of ocean waves rolling in to help you sleep!
Description: This is an 11-hour video (audio) of ocean rolling waves (like you're at the beach). This helps individuals improve sleep quality and can also help relax individuals.
Length: 11 hours
Ages: all ages

Title: Listen to this video of the rain in the rain forest in to help you sleep!
Description: This is an 8-hour video (audio) of rain in a forest (white noise) that aims to help produce 'deep sleep' and to relax the mind.
Length: 8 hours
Ages: all ages

Title: Listen to this video of sleep music to help you sleep!
Description: This is a 45-minute video (audio) of 'sleep music' to

help individuals fall asleep.
Length: 45 minutes
Ages: all ages

Title: Click here to read some helpful tips on how to improve your sleep!
Description: This is an informative website about sleep which offers 10 tips on how to improve sleep.
Length: N/A
Ages: 10 +

If you have questions, are experiencing difficulties, or need help with your behaviour change, please contact the kinesiologist using the contact information below:

Phone & E-mail

Phone: 613-737-7600 x 4003
Fax: 613-738-3908
cheoactive@cheo.on.ca

Business Hours

Monday – Friday
8.00 am – 4.00 pm
Weekend Closed

Sleep Pros and Cons

Play this activity below to learn a little more about changing your sleep habits! Read the sentence and decide whether or not you "agree" or "disagree" with the statement about changing your sleep habits. Once you have completed this, read the message that pops up that will support your response!

Activity	Agree/Disagree
**Getting the recommended amount of sleep can help me be healthier overall, if I am not sleeping enough right now.**	
**Getting the recommended amount of sleep can improve my mental health.**	
**Getting the recommended amount of sleep can improve my memory and attention.**	
**Getting the recommended amount of sleeping can help boost my daily mood.**	
**I'll need fewer naps during the day if I sleep better at night.**	
**Sometimes I will prioritize sleeping over other things happening at night, like staying up late watching a movie.**	
**Lying in bed awake when I can't sleep might make me feel anxious, uncomfortable, or bored.**	
**I might need to change my nighttime routine to change my sleep habits.**	
**Changing my sleep habits might be difficult and frustrating at first.**	
**I might have to change my eating habits to sleep better.**	
**I might have to start drinking less coffee or caffeinated drinks to sleep better.**	
**I might have to change my activity habits to sleep better.**	
**I won't use my phone close to bedtime if I change my sleep habits.**	
**I'll wake up at the same time every day, even on weekends.**	
**I will only use my bed for sleeping.**	
**I might have to take time away from schoolwork to sleep.**	

Show Reasons

If you have questions, are experiencing difficulties, or need help with your behaviour change, please contact the kinesiologist using the contact information below:

Phone & E-mail

Phone: 613-737-7600 x 4003
Fax: 613-738-3908
cheoactive@cheo.on.ca

Business Hours

Monday – Friday
8.00 am – 4.00 pm
Weekend Closed

Goals

Remember to make your goals SMART. See information making your goals SMART here

You have 4 active goals!

Desired Behaviour Change	Goal	View Plan	Mark Completed
I want to improve my physical activity habits	I Want to Make my Own Goal	PDF	
I want to improve my sleep habits	I Want to Make my Own Goal	PDF	
I want to swim more	Find fun games that will encourage me to go swimming!	PDF	
I want to reduce my screen time	I want to spend 10 minutes less watching TV/ videos online each day!	PDF	

Choose a new goal!

Filter By: Being Active Sleep Screen Time

Desired Behaviour Change	Goal	View Plan	Select Goal

You have completed 8 goals!

Desired Behaviour Change	Goal	View Plan	Reactivate Goal
I want to go to bed 15 minutes earlier than I usually do for one night	I want to go to bed 15 minutes earlier than I usually do for one night		
I want to make a to-do list for the week on Sunday night!	I want to make a to-do list for the week on Sunday night!		
I want to practice relaxing my muscles before bed once!	I want to practice relaxing my muscles before bed once!		
I Want to Learn Chair Yoga Poses	I Want to Learn Chair Yoga Poses		
I Want to Learn new Yoga Poses and Learn the Sun Salutation sequence	I Want to Learn new Yoga Poses and Learn the Sun Salutation sequence		

Desired Behaviour Change	Goal	View Plan	Reactivate Goal
Learn and practice some ball games with my family and/or friends!	Learn and practice some ball games with my family and/or friends!		↺
I want to learn fun new activities to do with my family and/or friends that will get us moving!	I want to learn fun new activities to do with my family and/or friends that will get us moving!		↺
I want to journal before bed once a week!	I want to journal before bed once a week!		↺

If you have questions, are experiencing difficulties, or need help with your behaviour change, please contact the kinesiologist using the contact information below:

Phone & E-mail

Phone: 613-737-7600 x 4003
Fax: 613-738-3908
cheoactive@cheo.on.ca

Business Hours

Monday – Friday
8.00 am – 4.00 pm
Weekend Closed

CHEO RESEARCH INSTITUTE
INSTITUT DE RECHERCHE

I WANT TO GO TO SLEEP A BIT EARLIER THAN I DO RIGHT NOW

GOAL

Go to bed 15 minutes earlier than my usual bedtime for one night.

When trying to make a change in your sleep routine, it's important to plan in advance and adjust slowly. Research recommends that you shift your sleep schedule by just 15-minutes per day, rather than all at once. In this plan, you will work on changing your bedtime 15 minutes at a time!

Remember that there is no time limit for how long it should take you to finish each step! **Be sure to go at your own pace**. Some steps may only take a day or two, others may take a week or more. Once you've finished a step, move on to the next one when you feel comfortable! If you're not ready, feel free to repeat the same step until you feel that it is easy to do and you're ready to move on.

If you wish, you can refer back to the **chart below** to track when you complete each step and see what you have accomplished!

**Step One**	Accomplished? ☐
**Step Two**	Accomplished? ☐
**Step Three**	Accomplished? ☐
**Step Four**	Accomplished? ☐
**Step Five**	Accomplished? ☐
**Step Six**	Accomplished? ☐

You can also at any time refer back to the Traffic Light page, where you can learn to recognize your body's cues. You will find the Traffic Light Page on the **Plan** page for physical activity, sleep or screen time. Use this Traffic Light page whenever you want to check in to see how you are feeling during these activities. Keeping yourself with a "green light" will help you to enjoy and feel comfortable and relaxed during each step.

Are you experiencing roadblocks or finding it hard to be successful?
Head to the "**Dealing with Roadblocks**" page to find some helpful tips!

STEP 1
Estimate how long it takes you to get ready for bed.

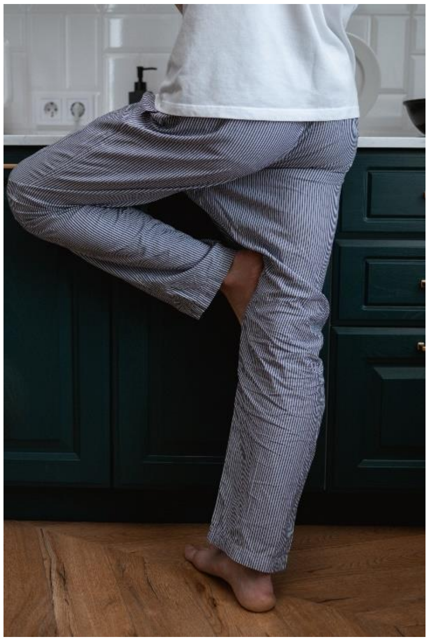

If you don't know already, **keep track of this over the next week to help you figure out approximately how long it takes you to get ready for bed**. Use a timer on your phone or computer. You can even have your family or friends track their bedtime routine length with you! You will use this information in Step 4.

Length of my nighttime routine:

If you don't have a nighttime routine and would like to create one, check out the plan for **creating a nighttime routine**. An example of steps you could include in your nighttime routine could be **brushing your teeth, showering, putting on your pjs, reading, and then going to sleep.**

Use this table to track the steps you take in your night time routine, and how long they took you!

Night Time Routine Step	How long did this take me?

Goal accomplished ☐

STEP 2
Identify distractions in your room or daily actions that might keep you awake at night.

Distractions can include **using your phone close to bedtime or having too much light or noise** in your room. Your actions from earlier in the day can also keep you up at night, for example, **exercising, consuming caffeine or nicotine, eating large meals, and napping too close to bedtime** can also disrupt your sleep.

Things that keep me up at night:

○
○
○
○

Work on eliminating one distraction at a time.

Goal accomplished ☐

Are you experiencing roadblocks or finding it hard to be successful?
Head to the "**Dealing with Roadblocks**" page to find some helpful tips!

STEP 3
Before bed, plan a relaxing nighttime activity to do if you can't fall asleep after 20 minutes.

Consider one of these activities: Reading, coloring, listening to music, or drinking a warm herbal tea (make sure it's caffeine free). **You can also check out our plans on journaling or mindfulness exercises! Be sure to keep the lights low and avoid any electronic devices that will keep you awake!**

Set up everything you need for this activity before you go to bed. For example, if you plan on reading, set out your book, a blanket, and house slippers before you go to bed. Complete these activities until you feel ready to fall asleep. These activities should be incorporated into your night time routine, so you can eventually fall asleep 15 minutes earlier.

Your turn!

**My relaxing nighttime activity:**<br>**For this activity I will need:**<br>○<br>○<br>○

From now on, if you haven't fallen asleep after lying in bed for **20 minutes**, be sure to move to another room to do the activity you planned. **Stay there until you feel really ready to sleep, not just tired**. You can try to look out for cues such as yawning, heavy eyelids, nodding off, etc. Practicing this habit will help teach your brain to think of your bed as a place that's only for sleep!

Goal accomplished ☐

Are you experiencing roadblocks or finding it hard to be successful?
Head to the "**Dealing with Roadblocks**" page to find some helpful tips!

STEP 4

Try going to bed 15 minutes earlier than usual for one night.

Current Bedtime:
New bedtime *(subtract 15 minutes from 'current bedtime')*:

Now that you know how long your nighttime routine takes *(Step 1)*, you can figure out when you should start getting ready for bed. Subtract the number you wrote down in Step 1 from your 'new bedtime'. **Write it down in the box below and try to plan your evening according to this time**.

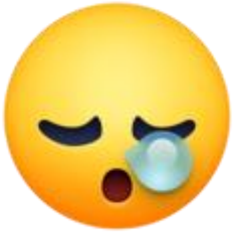

Start getting ready for bed: PM

Try to go to sleep at your "new bedtime" for one night. Changing your bedtime might be hard at first. **Remember, if you can't fall asleep sleep after 20 minutes, you should move to another room and do the nighttime activity that you planned in Step 3 until you are ready to sleep!**

Reflect: How did this go? Were you able to fall asleep close to the time you went to bed? Can you identify anything that kept you awake? What changes can you make?

Goal accomplished ☐

Are you experiencing roadblocks or finding it hard to be successful?
Head to the "**Dealing with Roadblocks**" page to find some helpful tips!

STEP 5

Once you've finished Step 5, try going to bed at your 'new bedtime' for more nights of the week!

You can **work on this step for multiple weeks** by slowly adding one more day to your goal each week or you can try to accomplish these goals in less time if you feel ready! Come back and check each box off as you go!

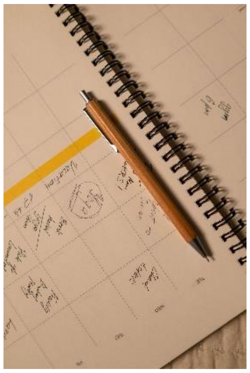

I went to bed 15 minutes earlier than usual for….

- ☐ 2 days/week ☐
- ☐ 3 days/week ☐
- ☐ 4 days/week ☐
- ☐ 5 days/week ☐
- ☐ 6 days/week ☐
- ☐ 7 days/week ☐

You don't need to stick to your new bedtime every single day before moving on to the next step. Just **aim for most days of the week and focus on building a new habit. Once you feel like you're used to your new bedtime, move on to step 7.**

Are you experiencing roadblocks or finding it hard to be successful?
Head to the "**Dealing with Roadblocks**" page to find some helpful tips!

STEP 6

Repeat steps 5 and 6 with a bedtime that is another 15 minutes earlier until you've reached your bedtime goal.

For a teenager, your bedtime should ideally allow you to get **8-10 hours of sleep** on a typical day. **Choose a bedtime goal that is realistic and manageable for you.**

Bedtime goal:

Goal accomplished ☐

GREAT JOB!

You've reached the end of the plan!
Head back to the CHEO active website (www.cheoactive.ca)
to keep learning about healthy lifestyles!

I WANT TO SWIM MORE!

Goal

I want to learn fun games that will encourage me to go swimming regularly!

There is no time limit for how long it should take you to finish each step!
Be sure to go at your own pace.

TIP!! Try practicing these movements outside of the pool first, or in front of family to increase your confidence in your movements!

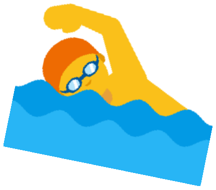

Things to remember!

- Pick an activity that you like doing and have fun in creating new ways to play it.
- Pick a space that will be safe, comfortable, and encouraging!
  Having a place where you feel safe, supported by those around you, and comfortable to take on a new challenge will make playing a lot more fun.

If you wish, you can refer back to the **chart below** to track when you complete each step and see what you have accomplished!

**Step One**	Accomplished? ☐
**Step Two**	Accomplished? ☐
**Step Three**	Accomplished? ☐
**Step Four**	Accomplished? ☐
**Step Five**	Accomplished? ☐
**Step Six**	Accomplished? ☐

You can also at any time refer back to the Traffic Light page, where you can learn to recognize your body's cues. You will find the Traffic Light Page on the **Plan** page for physical activity, sleep or screen time. Use this Traffic Light page whenever you want to check in to see how you are feeling during these activities. Keeping yourself with a "green light" will help you to enjoy and feel comfortable and relaxed during each step.

STEP 1: Learning different swimming strokes

Try learning different swimming strokes in the pool! You can even practice some of the movements outside of the pool first! Choose one or more of these movements and practice them. Once you feel like you have completed one movement, try out another!

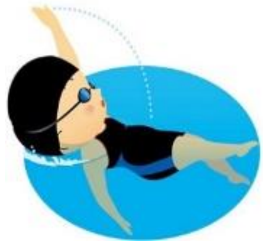

Technique 1: Backstroke – Start floating in the water face-up. You will be moving your arms in circular motions like a windmill in and out of the water. Kick your legs up and down to also help move yourself forward in the water.

Technique 2: Breaststroke – Start floating on your stomach with your face in the water and your arms straight above your head. The movement sequence will be: pull, breathe, kick, glide. Pull your body through the water by moving your arms from above your head to your sides in a circular motion. Next, lift your head out of the water and take a breath. After you take a breath, bend your knees and then whip your feet outwards in a circular motion until they are straight again and glide!

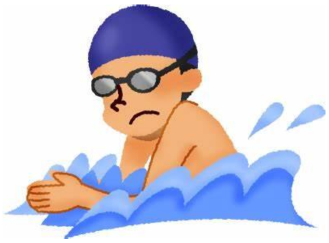

Technique 3: Butterfly – Start again by floating in the water face-down. Push your head down and forward in the water while bringing your hips up to the surface of the water to propel your body forward. While your hips are up, bend and straighten your knees (kick) to propel your head upwards to breath. As you breathe lift both arms out of the water beside your body and then swing them around to put them in the water in front of you. Your body's movement should look sort of like a wave!

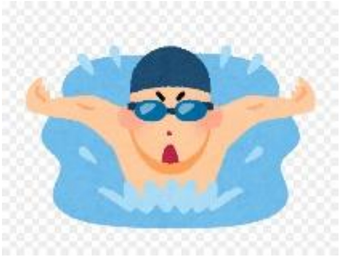

STEP 1 Continued: Learning different swim techniques

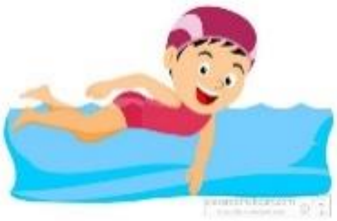

Technique 4: Freestyle – This stroke is very similar to a backstroke – except, you will be lying on your stomach! Start face-down in the water and kick your legs up and down continuously. Move your arms like a windmill out of the water (and back in the water) in a circular motion to propel yourself forward. Don't forget to breathe regularly while you move your arms!

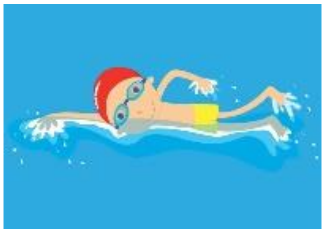

Technique 5: Sidestroke – Lay on your side in the water with your arm extended on the side you're laying on. Flutter kick your legs while you propel yourself forward with the arm on the surface of the water.

Want to see these techniques in action? Check out the swimming videos on this website: https://www.openfit.com/common-swimming-strokes

Goal accomplished ☐

STEP 2: Races

Grab your family or some friends to race against! Race while practicing different swimming techniques from STEP 1to find out which technique is your favorite, and which one makes you the fastest!

You can change up the races by making different rules for each race. Here are some race examples:

1. Race one length of the pool using the Butterfly Technique
2. Race two lengths of the pool using the Backstroke Technique
3. Race two lengths of the pool using the Sidestroke Technique for one length, and the Freestyle Technique for the other length back

Goal accomplished ☐

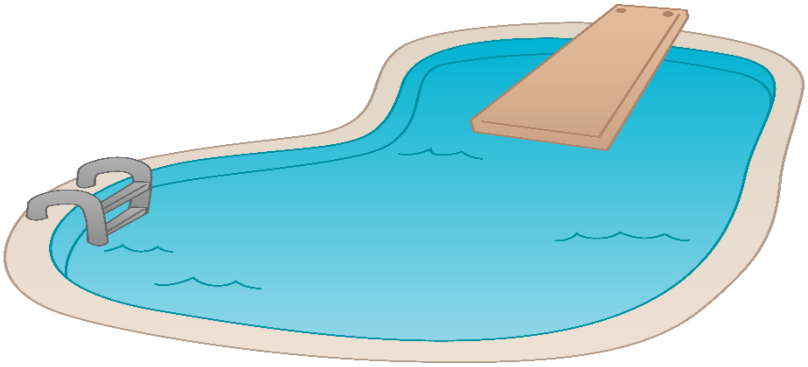

STEP 3: Ball race

Have a ball race with a family member or friend! Try pushing a ball in the water from one side of the pool to the other without throwing the ball. The first person to make it to the other side of the pool with their ball wins!

You can use any floaty toy if you do not have a ball!

Tip: Try out different swimming techniques to see which is the easiest to push the ball.

Goal accomplished ☐

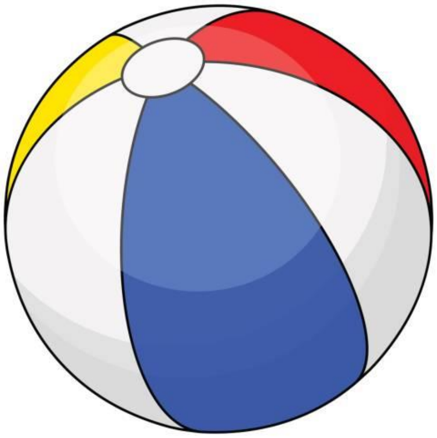

STEP 4: Marco Polo!

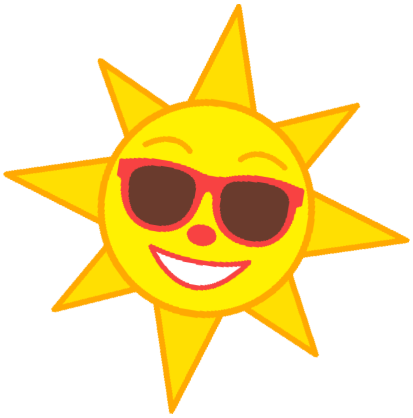

Play this game with friends or family on the next hot day in the pool or at the beach!

1. Designate one player as 'Marco' and all the other players as 'Polo'. The player who is Marco will be "it".
2. The goal of the game is to not get caught by Marco.
3. Marco will have their eyes closed and will be trying to catch the Polos. All Polos will have their eyes open
4. Marco will yell "Marco!" and all other players must yell "Polo!" back.
5. Marco will then try to catch the other players by listening to where their screams are coming from.
6. If a Polo is tagged, they become Marco.
7. Continue playing this game until everyone has had a turn being Marco!

Goal accomplished ☐

STEP 5: Review week!

You are halfway through the plan!! Way to go!

For this step, go back in the plan and pick your FAVORITE activity so far! Do that one again!

TIP!! Grab a friend or family member and get them to do that step with you! Make a competition to see which one can check more items off on the scavenger hunt or see what their favorite songs are!

Goal accomplished ☐

STEP 6: Ping-Pong ball hunt

Start this fun game by grabbing a bag of Ping-Pong balls and toss them randomly in the pool. Have someone outside of the pool start a timer to see how long it takes you to gather all of the balls!

You can also use different water-safe objects in the pool to increase the difficulty.

Goal accomplished ☐

Tip: Try this activity on your own or compete against a friend or family member to see who can collect the most Ping-Pong balls or desired objects!

TIP!! Practice getting the Ping-Pong by using different swimming techniques that you learned in Step 1!

STEP 7: Sharks and minnows

Grab a group of friends or family members to play Sharks and Minnows!

1. Designate one person to be the Shark and everyone else will be the Minnows.
2. The Shark stands in the middle of the pool, and the Minnows stand on the outer edges of the pool.
3. When the Shark yells "GO" the Minnows try to swim past the Shark to the other side of the pool without getting tagged by the shark.
4. If a Minnow is tagged by the Shark, they too become a Shark!
5. The game ends once there is only one Minnow left who has not been tagged by the Sharks.

Tip: It gets harder and harder each time as more Minnows become Sharks, try to strategize ways to avoid the Sharks by using different swimming techniques!

Goal accomplished ☐

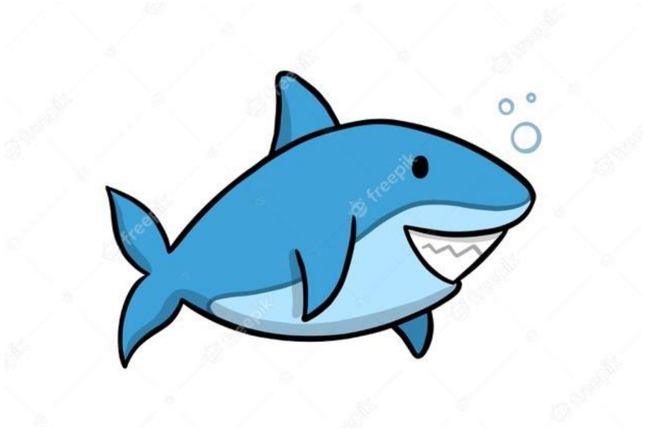

STEP 8: Quizmaster

Grab a friend or family member to play this game!

One person is in the center of the swimming pool, and the other player is outside of the pool waiting to jump in. The player in the pool is the designated Quizmaster. The Quizmaster will come up with a question to ask the other player while they're on the edge of the pool. The player who is jumping into the pool needs to answer the Quizmaster's question while they're in the air before they hit the water!

Here are some questions you can ask:

1. How many legs does a spider have?
2. What do caterpillars turn into?
3. What do bees make?
4. How many days are in a year?
5. What country are we in?
6. What is the fastest animal?
7. What planet do we live on?
8. What's your favorite holiday?
9. What are your parents' names?
10. How many colors are in a rainbow?
11. What day comes after Tuesday?
12. How many letters are in the alphabet?
13. What country is New York City in?
14. What color is your swimsuit?
15. What is a color that starts with the letter G?
16. How many days are in a week?
17. What is a word that starts with a C?
18. What is the capital of the country we're in?
19. What language are we speaking?
20. What country is Paris in?

Goal accomplished ☐

STEP 9: Follow the leader

Grab your family or friends to play this game in the pool!

Have one person be the designated 'leader' and the others are the 'followers'! The leader can do any water-related thing they want, and the followers must imitate what the leader does!

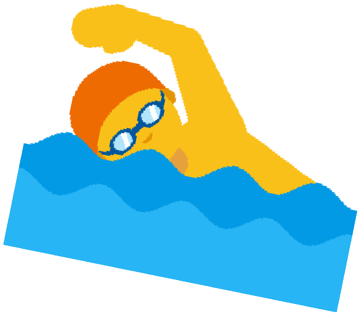

Here are some examples:
1. The leader does the Backstroke Technique for 5 seconds!
2. The leader jumps into the pool!
3. The leader does a handstand under water!

Goal accomplished ☐

STEP 10: Review week!

You made it to the end of the plan!! Way to go!

For this step, go back in the plan and pick your FAVORITE activity so far! Do that one again!

TIP!! Grab a friend or family member and get them to do that step with you! Make a competition to see which one can check more items off on the scavenger hunt or see what their favorite songs are!

Goal accomplished ☐

I WANT TO REDUCE MY SCREEN TIME

GOAL

Each day I want to spend 10 minutes less watching TV or videos online.

Electronics are a normal part of our lives and can be great for learning, having fun and helping us stay connected with friends and family. However, spending a lot of time using screens in ways that don't benefit can harm your physical and mental health. Screen time can include electronic devices such as using your phone or tablet, watching TV or playing video games. It can be difficult to reduce your screen time if you're in the habit of using these devices every day. In this plan you will work on gradually reducing the time you spend watching TV or online videos in a day.

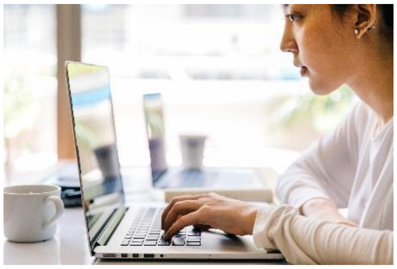

Remember that there is no time limit for how long it should take you to finish each step! **Be sure to go at your own pace**. Some steps may only take a day or two, others may take a week or more. Once you've finished a step, move on to the next one when you feel comfortable! If you're not ready, feel free to repeat the same step until you feel that it is easy to do and you're ready to move on.

If you wish, you can refer back to the **chart below** to track when you complete each step and see what you have accomplished!

**Step One**	Accomplished? ☐
**Step Two**	Accomplished? ☐
**Step Three**	Accomplished? ☐
**Step Four**	Accomplished? ☐
**Step Five**	Accomplished? ☐
**Step Six**	Accomplished? ☐
**Step Seven**	Accomplished? ☐

Are you experiencing roadblocks or finding it hard to be successful?
Head to the "**Dealing with Roadblocks**" page to find some helpful tips!

You can also at any time refer back to the Traffic Light page, where you can learn to recognize your body's cues. You will find the Traffic Light Page on the **Plan** page for physical activity, sleep or screen time. Use this Traffic Light page whenever you want to check in to see how you are feeling during these activities. Keeping yourself with a "green light" will help you to enjoy and feel comfortable and relaxed during each step.

Are you experiencing roadblocks or finding it hard to be successful?
Head to the "**Dealing with Roadblocks**" page to find some helpful tips!

STEP 1

Replace 10 minutes of your TV time with a more active form of entertainment.

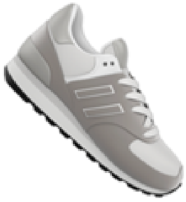

Try exchanging some of your TV time for a walk while you listen to a podcast or music. You can also try to make some of your TV time a bit more active by doing some chores, standing, dancing or doing some light physical activity while watching.

Write down your plan below. *Example*: *I will replace 10 minutes of "The Office" with 10 minutes of listening to a podcast while walking*. ***Your turn!***

I will…

Use this tracking chart below to **track what activities you replaced TV time** with:

TV Show/ Movie I would Normally Watch	Activity I Replaced it With	Amount of Time I Spent Doing Activity:
Example: Friends	Example: Walk Outdoors	Example: 15 Minutes

If you are successful with this step for one day, **try repeating it more often**!

Goal accomplished ☐

Are you experiencing roadblocks or finding it hard to be successful?
Head to the "**Dealing with Roadblocks**" page to find some helpful tips!

STEP 2
Start tracking your video streaming time.

It's easy to spend a lot of time watching TV shows, movies, and YouTube channels that you like. Tracking your video streaming time is the first step in starting to reduce it!

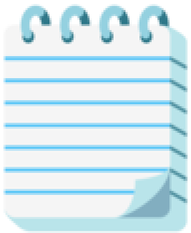

Grab a piece of paper or make a note on your phone. **Create a TV/video log** and fill it in for a week. Every time you sit down to watch something, write down when you start watching and when you stop watching. At the end of the week, add up all of your entries to get your '**weekly video streaming time**'. Divide this number by 7 to get your '**average daily video streaming time**'. You will use these times to help you set gaming limits in Step 6!

You can use the chart below to track your video/ tv log:

**Day of the Week**	**When you Started Watching**	**When you Stopped Watching**	**TV/ Movie Watched**
Monday			
Tuesday			
Wednesday			
Thursday			
Friday			
Saturday			
Sunday			

My weekly video streaming time:
My average daily video streaming time:

Reflect: Start to think about how much you want to reduce this number by.

Goal accomplished ☐

Are you experiencing roadblocks or finding it hard to be successful?
Head to the "**Dealing with Roadblocks**" page to find some helpful tips!

STEP 3

Disable auto-play on your streaming services and/or on YouTube.

Auto-play makes it easy to spend more time watching videos than you had originally planned. Turning off auto-play can give you a chance to think and make a choice about whether you want to keep watching.

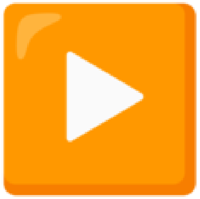

For Netflix:

- Sign in and go to the Your Account page
- Scroll down to "Profiles and Parental controls" and click your profile
- Click "Playback settings"
- Under Preferences, uncheck the box next to "Play next episode automatically"

For YouTube:

- Go to the watch screen of any video.
- At the bottom right of the video player, click the Auto-play switch to set it to "Off"

For Prime Video:

- Sign in and click your name at the top right of the page
- Click "Account settings"
- Click "Player"
- Change your auto-play setting to "Off"

Goal accomplished ☐

Are you experiencing roadblocks or finding it hard to be successful?
Head to the "**Dealing with Roadblocks**" page to find some helpful tips!

STEP 4

Practice stopping your show or video when the action slows down.

Some parts of TV shows or movies are less interesting than others. To get out of the habit of needing to watch something all the way through, look for these natural breaks in the action. Take this as an opportunity to **pause what you're watching and come back to it another time**!

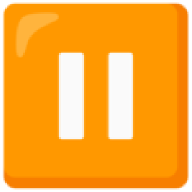

Reflect: Did this strategy help you spend less time watching TV/videos?

Keep practicing this step as you move on!

Goal accomplished ☐

Are you experiencing roadblocks or finding it hard to be successful?
Head to the "**Dealing with Roadblocks**" page to find some helpful tips!

STEP 5

Make TV/videos a reward for finishing other tasks.

You don't need to stop watching TV or online videos completely but making yourself earn an episode can help you reduce your screen time and make watching feel like a special reward.

Make a deal with yourself to reward yourself with an episode of a show or a YouTube video, if you do something else first. For example, you could choose cleaning your room or finishing your homework.

Example: *I can watch one episode of Grey's Anatomy if I finish doing my laundry*. **Your turn!**

Goal accomplished ☐

Are you experiencing roadblocks or finding it hard to be successful?
Head to the "**Dealing with Roadblocks**" page to find some helpful tips!

STEP 6
Set a daily limit on your video streaming time.

Start with a longer time limit that you know you can stick to. To create your first daily limit, start by subtracting 10 minutes from your 'average daily video streaming time' *(Step 2)*.

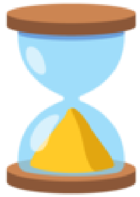

My daily video streaming limit:

If you can stick to this limit for one day, **try to repeat this step more often!**

TIP! When you sit down to watch something, set a timer on your phone to help you keep track. Once you've reached your daily limit, do your best to turn off what you're watching.

Goal accomplished ☐

Are you experiencing roadblocks or finding it hard to be successful?
Head to the "**Dealing with Roadblocks**" page to find some helpful tips!

STEP 7

Once you've finished step 6, try to keep reducing your video streaming time!

You can **work on this step for multiple weeks** by gradually reducing your daily video streaming limit by another 10 minutes each week. Repeat this step until you've reached a video streaming time that you're happy with. Continue filling in your TV/video log to help you keep track of your progress!

Remember, it is recommended that keeping your total recreational screen time to 2 hours or less per day with optimize your physical and mental health!

Recreational screen time includes all apps, TV shows, movies, video/computer games and more, but does not include screen time for school, work, homework or connecting with family and friends.

As you reduce your screen time, **try to use this time to do other healthy activities** such as exercising, practicing a skill or hobby, spending time with friends or family, or even sleeping more!

You can use the log below to track your video/ tv log throughout the weeks:

Day of the Week	When you Started Watching	When you Stopped Watching	TV/ Movie Watched
Monday			
Tuesday			
Wednesday			
Thursday			
Friday			
Saturday			
Sunday			

Are you experiencing roadblocks or finding it hard to be successful?
Head to the "**Dealing with Roadblocks**" page to find some helpful tips!

TIP! Here is a list of activities that you can do instead of watching videos online:

- Going for a walk or hike
- Playing a sport with friends, family or by yourself
- Exercising
- Practicing yoga
- Journaling or doing mindfulness exercises
- Cooking/baking
- Painting or drawing
- Reading
- Playing an instrument
- Connecting with friends or family

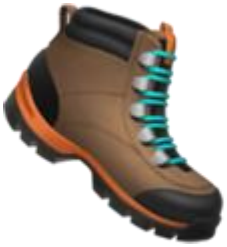

Check out the plans on the "**About physical activity**" and "**About sleep**" pages to help you get started with some of these activities!

Goal accomplished ☐

GREAT JOB!

You've reached the end of the plan!
Head back to the CHEO active website (www.cheoactive.ca)
to keep learning about healthy lifestyles!

Traffic light – Learn to recognize your body's cues!

Traffic lights help us drive safely and navigate the roads without having any accidents or injuries. We will use the traffic light system during this program to help keep you safe, comfortable and relaxed.

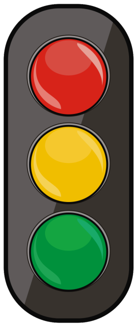

Red means STOP. Listen to what your body is telling you. Are your muscles in pain? Are you out of breath? Are you feeling anxious because this activity is too much? All of these signals would tell you to stop and safely

Yellow means SLOW DOWN! You are noticing some signs that you are heading towards the red zone. This could be aches in your muscles, breathing heavier, feeling unsure about your activity, and more. Slow down and work on getting back to the green zone.

Green means GO! You are feeling confident, comfortable, and ready to take on the activity.

Take a minute to think about your green zone. What tells you that you are ready to do this activity?

- Example: I am confident, hydrated, well rested, energized
-
-
-

Now, take a minute to think about your red zone. What tells you that you need to stop and take a break? Or that this is not an activity that is comfortable for you and your body?

- Example: pain, feeling out of breath, tired, anxious, hungry
-
-
-

During each step you will learn to pay attention to the signals that your body is giving you. After each step, you can write down which zone you were in and what signals told you that you were in that zone.

There is no time limit for how long it should take you to finish each step! **Be sure to go at your own pace.** Once you've finished a step and feel comfortable with it, move on to the next one. Keep in mind that these steps can be repeated as many times as you need in order to feel comfortable moving onto the next step.

Click here to download and print off the Traffic Light page

If you have questions, are experiencing difficulties, or need help with your behaviour change, please contact the kinesiologist using the contact information below:

Phone & E-mail

Phone: 613-737-7600 x 4003
Fax: 613-738-3908
cheoactive@cheo.on.ca

Business Hours

Monday – Friday
8.00 am – 4.00 pm
Weekend Closed

Dealing with Roadblocks

Click on each of the roadblocks below to learn more.

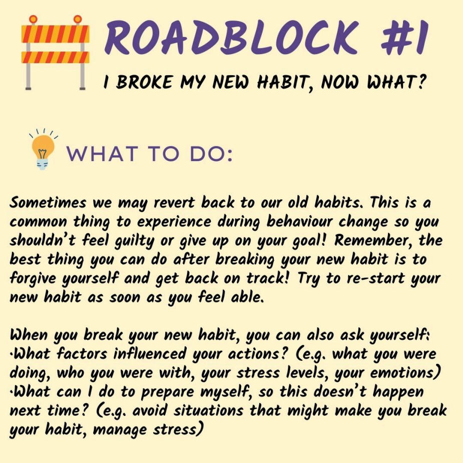

[Road Block #1: I broke my new habit, now what?]

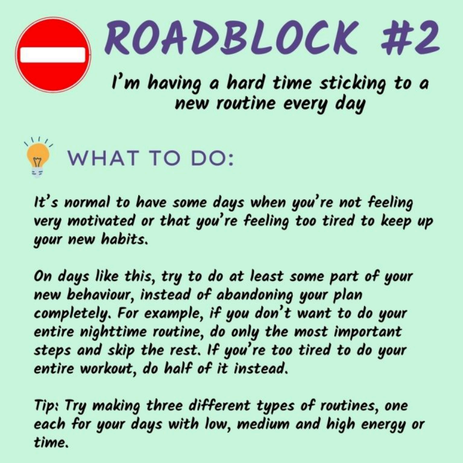

[Road Block #2: I'm having a hard time sticking to a new routine every day]

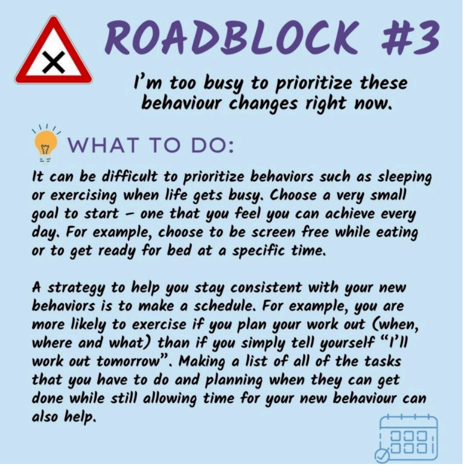

[Road Block #3: I'm too busy to prioritize these behaviour changes right now]

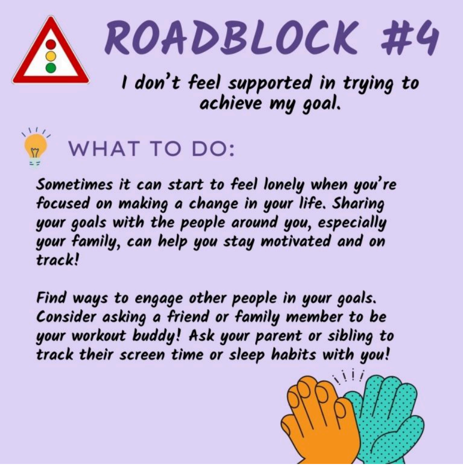

[Road Block #4: I don't feel supported in trying to achieve my goal]

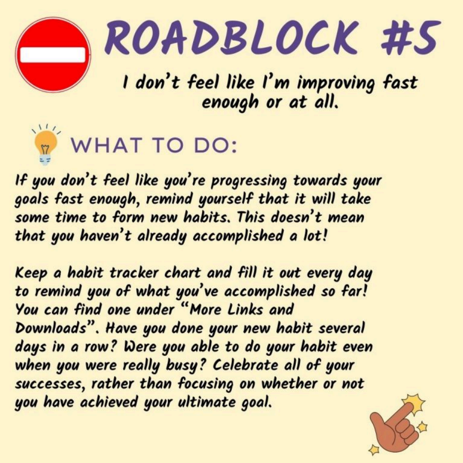

[Road Block #5: I don't feel like I'm improving fast enough or at all.]

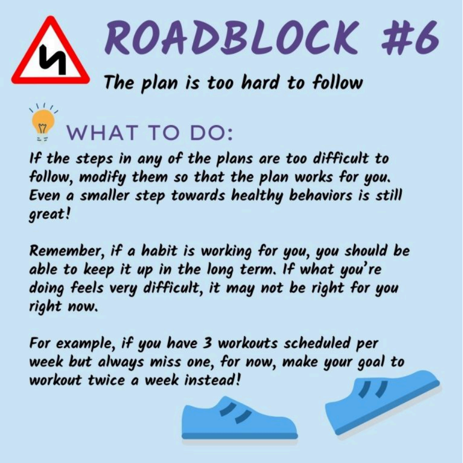

[Road Block #6: The plan is too hard to follow]

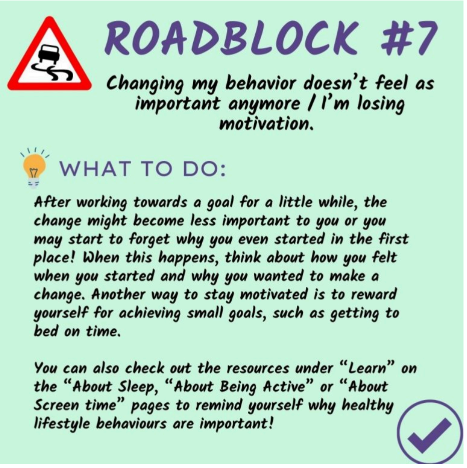

[Road Block #7: Changing my behavior doesn't feel as important anymore / I'm losing motivation.]

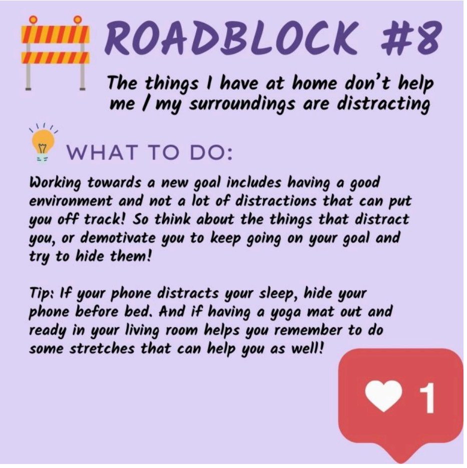

[Road Block #8: The things I have at home don't help me / my surroundings are distracting]

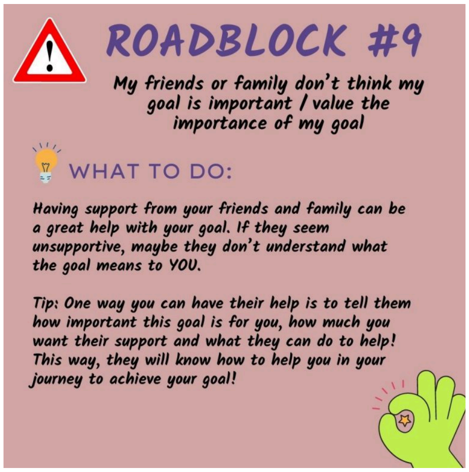

[Road Block #9: My friends or family don't think my goal is important / value the importance of my goal]

If you have questions, are experiencing difficulties, or need help with your behaviour change, please contact the kinesiologist using the contact information below:

Phone & E-mail

Phone: 613-737-7600 x 4003
Fax: 613-738-3908
cheoactive@cheo.on.ca

Business Hours

Monday – Friday
8.00 am – 4.00 pm
Weekend Closed
